# The effect of prenatal balanced energy and protein supplementation on gestational weight gain: An individual participant data meta-analysis in low- and middle-income countries

**DOI:** 10.1371/journal.pmed.1004523

**Published:** 2025-02-03

**Authors:** Dongqing Wang, Uttara Partap, Enju Liu, Janaína Calu Costa, Ilana R. Cliffer, Molin Wang, Sudeer Kumar Nookala, Vishak Subramoney, Brittany Briggs, Imran Ahmed, Alemayehu Argaw, Shabina Ariff, Nita Bhandari, Ranadip Chowdhury, Daniel Erchick, Armando García-Guerra, Masoumah Ghaffarpour, Giles Hanley-Cook, Lieven Huybregts, Fyezah Jehan, Fatemeh Kaseb, Nancy F. Krebs, Carl Lachat, Tsering Pema Lama, Dharma S. Manandhar, Elizabeth M. McClure, Sophie E. Moore, Ameer Muhammad, Lynnette M. Neufeld, Andrew M. Prentice, Amado D. Quezada-Sánchez, Dominique Roberfroid, Naomi M. Saville, Yasir Shafiq, Bhim P. Shrestha, Bakary Sonko, Sajid Soofi, Sunita Taneja, James M. Tielsch, Laéticia Céline Toe, Naser Valaei, Wafaie W. Fawzi

**Affiliations:** 1 Department of Global and Community Health, College of Public Health, George Mason University, Fairfax, Virginia, United States of America; 2 Department of Global Health and Population, Harvard T.H. Chan School of Public Health, Harvard University, Boston, Massachusetts, United States of America; 3 Institutional Centers for Clinical and Translational Research, Boston Children’s Hospital, Boston, Massachusetts, United States of America; 4 Division of Gastroenterology, Hepatology and Nutrition, Boston Children’s Hospital, Harvard Medical School, Boston, Massachusetts, United States of America; 5 Department of Epidemiology, Harvard T.H. Chan School of Public Health, Harvard University, Boston, Massachusetts, United States of America; 6 Department of Biostatistics, Harvard T.H. Chan School of Public Health, Harvard University, Boston, Massachusetts, United States of America; 7 Cytel Inc., India on behalf of the Bill & Melinda Gates Foundation, Seattle, Washington, United States of America; 8 DVPL Tech, Dubai, United Arab Emirates; 9 Certara USA, Inc. on behalf of the Bill & Melinda Gates Foundation, Seattle, Washington, United States of America; 10 Aga Khan University, Karachi, Pakistan; 11 Department of Food Technology, Safety and Health, Ghent University, Ghent, Belgium; 12 Society for Applied Studies, New Delhi, India; 13 Department of International Health, Johns Hopkins Bloomberg School of Public Health, Baltimore, Maryland, United States of America; 14 Centro de Investigación en Nutrición y Salud, Instituto Nacional de Salud Pública, Cuernavaca, Mexico; 15 Colonia Santa María Ahuacatitlán, Cerrada Los Pinos y Caminera. Cuernavaca, Mexico; 16 Faculty of Nutrition Sciences and Food Technology, Shahid Beheshti University of Medical Sciences, Tehran, Iran; 17 Nutrition, Diets, and Health Unit, International Food Policy Research Institute, Washington, DC, United States of America; 18 Department of Pediatrics and Child Health, the Aga Khan University, Karachi, Pakistan; 19 Department of Paramedical, Shahid Sadoughi University of Medical Sciences, Yazd, Iran; 20 University of Colorado School of Medicine, Aurora, Colorado, United States of America; 21 Nepal Nutrition Intervention Project, Sarlahi, Kathmandu, Nepal; 22 Mother and Infant Research Activities (MIRA), Kathmandu, Nepal; 23 RTI International, Durham, North Carolina, United States of America; 24 Department of Women & Children’s Health, King’s College London, London, United Kingdom; 25 MRC Unit The Gambia at the London School of Hygiene and Tropical Medicine, Fajara, The Gambia; 26 VITAL Pakistan Trust, Karachi, Pakistan; 27 Food and Agriculture Organization of the United Nations (FAO), Rome, Italy; 28 Centro de Investigación en Evaluación y Encuestas, Instituto Nacional de Salud Pública, Cuernavaca, Mexico; 29 Faculty of Medicine, Namur University, Namur, Belgium; Belgian Health Care Knowledge Centre, Brussels, Belgium; 30 Institute for Global Health, University College London, London, United Kingdom; 31 Center of Excellence for Trauma and Emergencies and Community Health Sciences, The Aga Khan University, Karachi, Pakistan; 32 Global Advancement of Infants and Mothers (AIM), Department of Pediatric Newborn Medicine, Brigham and Women’s Hospital, Harvard Medical School, Boston, Massachusetts, United States of America; 33 Harvard Humanitarian Initiative, Harvard T.H. Chan School of Public Health, Harvard University, Boston, Massachusetts, United States of America; 34 Department of Global Health, Milken Institute School of Public Health, George Washington University, Washington, DC, United States of America; 35 Nutrition and Metabolic Diseases Unit, Health Sciences Research Institute (IRSS), Bobo-Dioulasso, Burkina Faso; 36 Department of Nutrition, Harvard T.H. Chan School of Public Health, Harvard University, Boston, Massachusetts, United States of America; Makerere University College of Health Sciences, UGANDA

## Abstract

**Background:**

Understanding the effects of balanced energy and protein (BEP) supplements on gestational weight gain (GWG) and how the effects differ depending on maternal characteristics and the nutritional composition of the supplements will inform the implementation of prenatal BEP interventions.

**Methods and findings:**

Individual participant data from 11 randomized controlled trials of prenatal BEP supplements (*N* = 12,549, with 5,693 in the BEP arm and 6,856 in the comparison arm) in low- and middle-income countries were used. The primary outcomes included GWG adequacy (%) and the estimated total GWG at delivery as continuous outcomes, and severely inadequate (<70% adequacy), inadequate GWG (<90% adequacy), and excessive GWG (>125% adequacy) as binary outcomes; all variables were calculated based on the Institute of Medicine recommendations. Linear and log-binomial models were used to estimate study-specific mean differences or risk ratios (RRs), respectively, with 95% confidence intervals (CIs) of the effects of prenatal BEP on the GWG outcomes. The study-specific estimates were pooled using meta-analyses. Subgroup analyses were conducted by individual characteristics. Subgroup analyses and meta-regression were conducted for study-level characteristics. Compared to the comparison group, prenatal BEP led to a 6% greater GWG percent adequacy (95% CI: 2.18, 9.56; *p* = 0.002), a 0.59 kg greater estimated total GWG at delivery (95% CI, 0.12, 1.05; *p* = 0.014), a 10% lower risk of severely inadequate GWG (RR: 0.90; 95% CI: 0.83, 0.99; *p* = 0.025), and a 7% lower risk of inadequate GWG (RR: 0.93; 95% CI: 0.89, 0.97; *p* = 0.001). The effects of prenatal BEP on GWG outcomes were stronger in studies with a targeted approach, where BEP supplements were provided to participants in the intervention arm under specific criteria such as low body mass index or low GWG, compared to studies with an untargeted approach, where BEP supplements were provided to all participants allocated to the intervention arm.

**Conclusions:**

Prenatal BEP supplements are effective in increasing GWG and reducing the risk of inadequate weight gain during pregnancy. BEP supplementation targeted toward pregnant women with undernutrition may be a promising approach to delivering the supplements.

## Abstract 2

Using individual participant data, Dongqing Wang and colleagues find that prenatal balanced energy and protein supplements can increase gestational weight gain.

## Author summary

**Why was this study done?:**

- Gestational weight gain has critical implications for maternal and child health.
- Balanced energy and protein supplements, dietary supplements where less than 25% of energy comes from protein, have shown beneficial effects on some pregnancy and birth outcomes.
- There is inconsistent evidence regarding the effects of balanced energy and protein supplements on gestational weight gain.

**What did the researchers do and find?:**

- Individual participant data from 11 randomized controlled trials of prenatal balanced energy and protein supplements in low- and middle-income countries were used, including data from 12,549 pregnant women.
- We found that the consumption of balanced energy and protein supplements during pregnancy increased gestational weight gain and reduced the risk of gaining inadequate weight during pregnancy.
- The effects of prenatal BEP on GWG outcomes were stronger in studies with a targeted approach, where BEP supplements were provided to participants with undernutrition, compared to studies with an untargeted approach, where BEP supplements were provided to all participants in the intervention arm.

**What do these findings mean?:**

- Prenatal balanced energy and protein supplements can prevent inadequate gestational weight gain, thereby leading to favorable pregnancy and birth outcomes.
- Given the practical constraints of scaling up prenatal balanced energy and protein supplementation, further efforts are needed to understand the effectiveness, cost-effectiveness, and implementation of different targeting strategies based on the individual characteristics of pregnant women.
- The limitations of this study included the exploratory nature of the subgroup analyses and the use of Institute of Medicine recommendations for gestational weight gain that were developed for high-income countries.

## Introduction

Gestational weight gain (GWG) has critical implications for the short- and long-term health of the mother and the offspring. On the one hand, women who gain inadequate weight during pregnancy have elevated risks of small-for-gestational-age (SGA) births [1–4], preterm births [1,3,5], low birthweight [1,2,4,5], and neonatal and infant death [6,7]. On the other hand, excessive GWG leads to higher risks of macrosomia [3], large-for-gestational-age births [3], severe maternal morbidity [8], and postpartum weight retention [9]. Emerging evidence suggests that excessive GWG also increases the risk of overweight and obesity in the future lives of the offspring [10]. Due to these health implications and its modifiable nature, GWG is increasingly recognized as a key indicator for monitoring the overall health of the pregnancy and a promising yet underutilized target for antenatal care [1].

Food insecurity, inadequate dietary intake, and maternal undernutrition are the main nutritional contributors to inadequate GWG [1]. It is critical to ensure appropriate energy and macronutrient intake and adequate micronutrient intake to meet maternal and fetal needs during pregnancy [11]. Several prenatal nutritional supplements can be used to accommodate the increased maternal nutritional needs during pregnancy [12]. A nutritional intervention that has attracted increasing interest is balanced energy and protein (BEP) supplements, which are dietary supplements where less than 25% of energy comes from protein [13]. BEP can take different forms, such as solid foods, beverages, and lipid-based nutrient supplements, and can be provided to pregnant women to supplement their home diets. BEP supplements can also incorporate essential minerals and vitamins to meet the increasing demands of micronutrients for pregnant women and their fetuses [14].

Previous studies have shown that prenatal BEP supplements reduce the risk of stillbirths and SGA births and increase birthweight [13,15–18]. However, evidence regarding the effect of BEP on GWG is mixed. A Cochrane systematic review reported that prenatal BEP supplementation was not significantly associated with the weekly rate of GWG yet noted that the finding was based on very-low-quality evidence [13]. Meta-analyses [16,19] showed that prenatal BEP supplements increased the weekly rate of GWG by 21 grams per week. The inconsistent body of literature may be due to the wide variation in the composition of BEP supplements, the heterogeneous study settings with different baseline food insecurity levels, different targeting strategies of BEP supplements, and methodological challenges related to other conventionally used measures of GWG [20].

Understanding the different effects of prenatal BEP supplements by maternal characteristics and nutritional composition informs the design and targeting of prenatal BEP interventions toward individual pregnant women who are more likely to benefit from prenatal BEP in resource-limited settings. In this analysis, we aimed to use individual participant data from randomized controlled trials in low- and middle-income countries (LMICs) to determine the effect of prenatal BEP supplements on GWG. We also evaluated how the effect of prenatal BEP supplements differs by maternal characteristics and nutritional composition of the supplements.

## Methods

### Ethics statement

The Harvard T.H. Chan School of Public Health Institutional Review Board determined this secondary analysis of existing data was not human participants research because all data had been deidentified prior to receipt, and no new data collection or human subject interaction were involved. Informed consent was therefore not considered applicable.

### Data source

This meta-analysis was conducted using individual participant data from randomized controlled trials of prenatal BEP supplementation in LMICs. We used data from the Gestational Weight Gain Pooling Project, an individual participant data meta-analytical project aimed to address knowledge gaps related to the distributions, determinants, and consequences of suboptimal gestational weight gain in LMICs. We have previously described the design and procedures of this pooling project [4,21,22]. We systematically searched the literature using PubMed, Embase, Web of Science, and the Cochrane Library from the inception of each database through June 3, 2021, to identify eligible studies. The inclusion criteria were:

1. Randomized controlled trials (RCTs), which could be individually randomized or cluster randomized;
2. Participants were pregnant at enrollment or enrolled before pregnancy and followed up in pregnancy;
3. Studies conducted in a low-income, lower-middle-income, or upper-middle-income economy defined by the World Bank country classification for the 2021 fiscal year;
4. A dietary supplement provided during pregnancy in which less than 25% of the energy is from protein. The supplement could take various formats, such as food rations, beverages, or lipid-based nutrient supplements. When the proportion of energy from protein was not directly reported, we calculated the proportion as grams of protein × 4 kcal / total amount of calories in kcal × 100%;
5. The dietary supplement could be provided alone or in combination with a co-intervention;
6. At least 1 group in the study did not receive a prenatal dietary supplement considered BEP.

Exclusion criteria of the search were:

1. Studies without any measures of maternal weight during pregnancy;
2. Studies conducted exclusively among women with preexisting health conditions, such as anemia, preeclampsia, human immunodeficiency virus infection, or diabetes;
3. Studies of small-quantity lipid-based nutrient supplements (SQ-LNS), which provide less than 120 kcal per day, were excluded as we have specifically examined the effects of SQ-LNS on GWG in detail elsewhere [21].

We also reviewed the references of the identified studies and previous systematic reviews [13,15–19] to identify additional studies of relevance. Two team members independently conducted the title and abstract screening, with any discrepancies resolved by discussion. One team member conducted the full-text screening. After the full-text screening, we contacted the corresponding authors of all identified studies to seek collaboration and data contribution. For those willing to participate, we worked with the principal investigators to pursue appropriate data-sharing agreements. As individual participant data became available, we worked to examine data completeness, map relevant variables, and harmonize the data across studies. This data-sharing and harmonization process was substantially supported by the Knowledge Integration team at the Bill & Melinda Gates Foundation. Data from 26 studies were sought, of which 12 provided individual participant data by the time of the analysis. Of these 12 studies, 11 were included in the final analysis, and one was excluded due to data issues preventing inclusion in the final analysis. The characteristics of the 11 studies included in the final analysis are shown in **Table 1** [23–33]. An updated systematic search was conducted on October 7, 2024, and no additional eligible studies were identified. The characteristics of the 15 non-included studies (14 due to lack of independent participant data and one due to data issues) are shown in **S1 Table**. This work was registered with the International Prospective Register of Systematic Reviews (PROSPERO ID: CRD42023380556). This study is reported as per the Preferred Reporting Items for Systematic Reviews and Meta-Analyses (PRISMA) guideline (**S2 Table**). The PRISMA flow diagram is shown in **S1 Fig**.

**Table 1:** Characteristics of the 11 studies included in the individual participant data meta-analysis of the effect of prenatal BEP supplements on GWG^1^.

Study	Country	Intervention	Comparison	Timing of intervention initiation	Daily energy content	Forms of BEP	Delivery strategy	GA measure
Kaseb, 2002	Iran	Traditional food supplements of 400 kcal and 15 g protein daily. Supplements were composed of rice-milk porridge, lentils, pottage, cheese, yogurt, eggs, and milk with bread, given 5 days a week. Supplements were delivered from the fourth month of pregnancy to childbirth	No supplementation	From 4 months of gestation	400 kcal	Food ration	Untargeted	Unclear; possibly LMP
Huybregts, 2009	Burkina Faso	72 g of MMN-fortified food supplement providing 1.56 MJ (372 kcal) and 14.7 g protein. The food supplement consisted of 33% peanut butter, 32% soy flour, 15% vegetable oil, 20% sugar, and UNIMMAP in powdered form.	MMS as the UNIMMAP formulation	Mean GA at enrollment around 16 weeks	372 kcal	Lipid-based supplement	Untargeted	Ultrasound
Moore, 2012^2^	The Gambia	1) BEP + IFA: A food-based supplement providing a comparable level of iron and folate to the IFA-only arm but with the addition of energy, protein, and lipids. The food supplement provided 746 kcal and 20.8 g protein. 2) BEP + MMS: A micronutrient-fortified, food-based supplement providing comparable levels of micronutrients to the MMS arm and energy, protein, and lipid content. The food supplement provided 746 kcal and 20.8 g protein	1) IFA with 60 mg iron and 400 μg folate, representing the usual standard of care during pregnancy. 2) MMS as the UNIMMAP formulation.	<20 weeks gestation	746 kcal	Lipid-based supplement	Untargeted	Ultrasound
Saville, 2018^3^	Nepal	PLA + 150 g Super Cereal (fortified wheat soya blend plus 10% sugar) per day, providing 570 kcal, with 17% from protein, and meeting most micronutrient requirements for pregnant women	1) Control; 2) PLA; 3) PLA + cash	>8 weeks gestation	570 kcal	Food ration	Untargeted	LMP
Hambidge, 2019^4^	Guatemala, India, Pakistan	Small-quantity LNS of 118 kcal and 2.6 g protein. Participants were also provided with an additional daily lipid-based protein-energy supplement if they had a BMI <20 at any time while receiving the small-quantity LNS or had weight gain in the second or third trimesters of pregnancy less than the IOM guidelines; if consumed completely, this additional supplement provided 300 kcal and 11 g protein (approximately 15% energy) without additional supplemental micronutrients	Standard of care (typically IFA)	12–14 weeks gestation	418 kcal	Lipid-based supplement	Targeted based on BMI and GWG	Ultrasound
Neufeld, 2019^3^	Mexico	Micronutrient-fortified foods providing 250 kcal and 12 g protein	1) MMS tablets; 2) micronutrient powder	<25 weeks gestation	250 kcal	Food ration	Untargeted	LMP
Khan, 2021^3^	Pakistan	A monthly ration of 5 kg wheat soya blend plus (165 g/d) was provided to women during pregnancy and for the first 6 months of lactation. A daily ration of supplements provided 633 kcal and 29.1 g protein	Routine standard of care	19% enrolled <12 weeks gestation, 55% enrolled 13–27 weeks gestation, and 25% enrolled ≥28 weeks	633 kcal	Food ration	Untargeted	LMP
Taneja, 2022^5^	India	Interventions in 4 domains: health, nutrition, psychosocial care and support, and WaSH during preconception, pregnancy, and early childhood. Weekly supplies of locally prepared snacks containing cereal, pulses, soya, oil, sugar, salt, and milk powder (210 kcal, 2 g protein in the second trimester, and 400 kcal, 21 g protein in the third trimester) were provided for daily consumption for women with BMI <25. All women were also given milk (180 ml, 70 kcal, 6 g protein) 6 days a week throughout pregnancy. Women with BMI <18.5 or inadequate GWG were provided one additional hot cooked meal 6 days a week (500 kcal, 20 g protein) until delivery	Routine antenatal care from governmental or private sources (including IFA supplements)	9–13 weeks gestation	280–970 kcal depending on trimester and GWG	Food ration	Targeted based on BMI and GWG	Ultrasound
de Kok, 2022^6^	Burkina Faso	LNS in the form of an energy-dense peanut paste fortified with MMNs. The 72 g daily supplement provided 393 kcal and comprised 36% lipids, 20% protein, and 32% carbohydrates. Protein came from soy (61%), milk (25%), and peanut (15%)	Standard of care (IFA)	<21 weeks gestation	393 kcal	Lipid-based supplement	Untargeted	Ultrasound
Muhammad, 2022^7^	Pakistan	Approximately 800 kcal/d and 16–21 g of protein in a day as a ready-to-use supplement	Provision of antenatal care, skilled birth attendants, nutrition counseling and IFA	8-<19 weeks gestation	800 kcal	Lipid-based supplement	Untargeted	Ultrasound
Erchick, 2023^6^	Nepal	A ready-to-eat snack in the form of lipid-based peanut paste packaged in individual sachets (72 g). Each sachet is a daily portion that provides calories (approximately 400 kcal), protein (approximately 14g), and multiple micronutrients at the estimated average requirement for pregnancy	Recommendation to enroll in ANC at a local health clinic and deliver at a certified birthing facility; nutrition, hygiene, breastfeeding, and infant care counseling; and a clean birthing kit. In pregnancy, women in both arms received IFA and albendazole if not provided via ANC	From 14 weeks gestation through the first 6 months of lactation	400 kcal	Lipid-based supplement	Untargeted	Ultrasound

^1^ ANC, antenatal care; BMI, body mass index; BEP, balanced energy and protein; GA, gestational age; GWG, gestational weight gain; IFA, iron and folic acid; IOM, Institute of Medicine; LMP, last menstrual period; LNS, lipid-based nutrient supplements; MMN, multiple micronutrients; MMS, multiple micronutrient supplements; MNP, micronutrient powder; PLA, PLA, participatory learning and action women’s groups; RDA, recommended daily allowance; RUSF, ready-to-use supplemental food; SP, sulfadoxine-pyrimethamine; UNIMMAP, UNICEF/WHO/United Nations multiple micronutrient supplements for pregnant and lactating women; WaSH, water, sanitation and hygiene.

^2.^ Randomized controlled trial with a factorial design. In the absence of a statistically significant interaction between interventions, the study arms were collapsed based on the provision of prenatal BEP.

^3^ Cluster randomized controlled trial.

^4^ The study arm that started supplementation from the preconceptional period was excluded as the analysis focused on the effect of prenatal supplements initiated during pregnancy. Data from the Democratic Republic of the Congo were also excluded due to missing gestational age data.

^5^ Randomized controlled trial with a factorial design. The 2 arms that included preconceptional intervention were removed because the analysis focused on prenatal supplements, and non-negligible statistical interaction was found between the effects of preconceptional and antenatal intervention on GWG.

^6^ The 2 postpartum arms were collapsed with the corresponding antenatal study arms based on the provision of prenatal BEP.

^7^ The intervention arm that provided oral azithromycin in addition to BEP and the intervention arm that provided oral nicotinamide and choline in addition to BEP were removed from the analysis, given the additional effects conferred by oral azithromycin and nicotinamide and choline.

We further applied individual-level criteria to identify eligible individual participants, including (1) singleton pregnancies (twins, triplets, quadruplets, and higher-order pregnancies were excluded whenever known); (2) availability of a pre-pregnancy or early-pregnancy weight collected during the first trimester, plus at least 1 weight measurement in the second or third trimesters; (3) in the absence of pre-pregnancy or early-pregnancy weight, availability of at least 1 weight measurement in the second trimester of pregnancy to enable the estimation of early-pregnancy gestational weight, the process of which was described below; (4) known gestational ages at the time of weight measurements; and (5) availability of maternal height measures. We included data from pregnancies that resulted in fetal loss (miscarriage and stillbirths) and maternal deaths.

### Estimation of early-pregnancy weight

We used pre-pregnancy weight measure (whenever available) to calculate GWG; in the absence of pre-pregnancy weight, we used first-trimester weight as a proxy for pre-pregnancy weight in GWG calculation. For women with neither pre-pregnancy nor early-pregnancy weight, we imputed their first-trimester weight. The details of this imputation model, including model development and validation, have been described elsewhere [34]. Briefly, we used mixed-effects models and restricted cubic splines to impute gestational weight at 9^0/7^ weeks of gestation using weights measured later during the second trimester of pregnancy. We imputed weight at 9 weeks to be consistent with the first available weight measure used in the INTERGROWTH-21^st^ Study, an international consortium that developed global GWG standards among normal-weight women [35]. Imputing the gestational weight at 9 weeks also reasonably balanced the degree of extrapolation (i.e., imputing values farther away from the center of the available data for studies without first-trimester weight). In the final analytical sample, GWG was calculated using the observed pre-pregnancy or first-trimester weight for 67.3% of the participants, whereas the remaining 32.7% relied on the imputed first-trimester weight in GWG calculation.

We calculated body mass index (BMI) by dividing pre-pregnancy (observed) or first-trimester weight (observed or imputed) in kilograms by the square of height in meters. For women aged ≥20 years, we used the World Health Organization (WHO) adult cutoffs to define underweight (BMI <18.5 kg/m^2^), normal weight (BMI: 18.5 to <25.0 kg/m^2^), overweight (BMI: 25 to <30.0 kg/m^2^), and obesity (BMI ≥30.0 kg/m^2^). For adolescent women (<20 years old), we used the WHO adolescent growth reference to define underweight (BMI-for-age Z-score: < -2), normal weight (BMI-for-age Z-score: -2 to < 1), overweight (BMI-for-age Z-score: 1 to < 2), and obesity (BMI-for-age Z-score: ≥2) [36].

### Study outcomes

The study outcomes included GWG percent adequacy at the last available weight measurement during pregnancy, estimated total GWG at delivery, and GWG adequacy within the second and third trimesters. We calculated the GWG percent adequacy as the ratio of the observed GWG to the weight gain recommended by the Institute of Medicine (IOM) [1]. First, we calculated the observed GWG as the difference between each weight measurement and the pre-pregnancy or early-pregnancy weight (observed or imputed), as shown in Eq 1.


ObservedGWG=weightatfollow‐upvisit–pre‐pregnancyorearly‐pregnancyweight [Eq 1]

Second, we estimated the IOM-recommended GWG at the time of each weight measurement, as shown in Eq 2.


$$Recommended\;GWG=\left(\frac{Expected\;T1\;GWG}{13.86\;weeks}\right)\times (13.86\;weeks-gestational\;age\;at\;first\;measured\;or\;imputed\;weight\;measure)+(gestational\;age\;at\;follow-up\;visit–13.86\;weeks)\times recommended\;weekly\;rate\;of\;GWG\;for\;T2\&T3$$ [Eq 2]

The expected first-trimester GWG was 2 kg, 1 kg, and 0.5 kg for underweight/normal weight, overweight, and obesity, respectively [37]. The IOM-recommended weekly rate of GWG in the second and third trimesters was 0.51 kg/week for underweight, 0.42 kg/week for normal-weight, 0.28 kg/week for overweight, and 0.22 kg/week for obesity [1].

Finally, we calculated the GWG percent adequacy (%) by dividing the observed GWG by the recommended GWG, as shown in Eq 3.


$$GWG\;percent\;adequacy\;(\%)=\frac{Observed\;GWG}{Recommended\;GWG}\times 100\%$$ [Eq 3]

GWG percent adequacy explicitly accounts for the gestational age at each weight measurement and takes advantage of the IOM recommendations [4,21,22,37–39]. We defined severely inadequate GWG at each visit as a percent adequacy less than 70%, inadequate GWG as a percent adequacy less than 90%, adequate GWG as a percent adequacy between 90% and 125%, and excessive GWG as a percent adequacy greater than 125%; these definitions accounted for the allowable range specified in the IOM recommendations and were consistent with the cutoffs used in previous studies [4,21,22,37–39]. We estimated the total GWG at delivery by multiplying the GWG percent adequacy at the last available weight measurement by the IOM-recommended GWG at delivery, which was calculated using each individual’s gestational age at delivery [21]. We calculated the trimester-specific weekly rates of weight gain using the first and last available measures within the second and third trimesters; inadequate and excessive rates of weekly GWG during the second and third trimesters were defined based on the lower and upper limits for the ranges specified in the IOM recommendations [1].

### Statistical analysis

We used a two-stage analytical approach to obtain the study-specific estimates from each study and combined the study-specific estimates using meta-analyses. Within each study, we used linear models to examine the effects of prenatal BEP supplements on GWG percent adequacy at the last weight measurement and the estimated total GWG at delivery, both as continuous outcomes. We used log-binomial models or modified Poisson models with robust variance estimation [40] to estimate the effects of prenatal BEP supplements on binary outcomes, including severely inadequate, inadequate, and excessive GWG at the last available weight measurement and inadequate and excessive weekly rate of GWG during the second and third trimesters. Log-binomial models allow for the direct estimation of risk ratios [41,42]. Compared to logistic models, log-binomial models are especially advantageous because odds ratios from logistic models are not valid approximations for risk ratios for outcomes with high incidence (e.g., greater than 10%), as was the case for the binary outcomes in this study [41,42]. Poisson models with robust variance estimation were used to handle the model convergence issue that occasionally occurred with the log-binomial models [40]. We assessed and confirmed model assumptions for all models. Specifically, in the linear models, we examined the linearity assumption using residual plots and the homoscedasticity assumption by plotting residuals against the fitted values plots. In the log-binomial and modified Poisson models, we assessed the goodness-of-fit using Pearson chi-square statistics and deviance residuals.

For cluster RCTs, we used generalized estimating equations with a compound symmetry correlation structure to account for clustering; an independence correlation structure provided similar results. For studies with multiple interventions other than prenatal BEP, such as a factorial design, we examined statistical interaction between prenatal BEP and the additional intervention by including product terms between the 2 interventions. We evaluated the significance of the interaction using likelihood ratio tests. In the absence of statistical interaction (statistical interaction defined as a *P*-for-interaction less than 0.10), we collapsed the intervention arms based on whether prenatal BEP supplements were provided. Among the 2 studies with a factorial design [26,30], statistically significant interaction was found in only 1 study between preconceptional and prenatal interventions [30]. As a result, the 2 arms that received preconceptional intervention were removed because the present analysis focused on the effects of prenatal interventions. We conducted intention-to-treat analyses using the randomly assigned treatment assignment as the independent variable.

In a sensitivity analysis, we adjusted for covariates including maternal age, maternal years of education, parity, gestational age at enrollment, maternal height, pre-pregnancy or early-pregnancy BMI, and hemoglobin concentration at enrollment, all as continuous variables. The availability of these covariates varied across studies, and the covariates available in the study were adjusted. These covariates were selected based on the literature on risk factors for inadequate and excessive GWG in LMICs [22, 43]. No covariates were excluded due to multicollinearity. We also conducted a sensitivity analysis where missing data on covariates were imputed using multiple imputation. We conducted multiple imputation using the fully conditional specification method using the PROC MI function in SAS. For each study, the imputation model included all covariates used in the analyses and the outcome variables. A total of 20 imputed data sets were generated for each study. Linear regression was used to impute the covariates as continuous variables. We used trace plots to confirm the convergence of the Markov chain Monte Carlo algorithm. The estimates from all 20 imputed data sets were pooled using the PROC MIANALYZE function in SAS to generate combined estimates. We used complete case analysis without covariate adjustment as the primary approach given the randomized designs that minimized confounding through random intervention allocation.

After obtaining the study-specific estimates, we used fixed-effect and random-effects inverse-variance meta-analyses to pool the study-specific effects using the DerSimonian and Laird method. We reported weighted mean differences (WMDs) with 95% confidence intervals (CIs) for continuous outcomes and risk ratios (RRs) and 95% CIs for binary outcomes. We assessed heterogeneity across studies using the I^2^ statistic, with thresholds of <30%, 30% to 60%, and >60% considered low, moderate, and high heterogeneity, respectively.

To identify subgroups of pregnant women for which prenatal BEP supplements may have greater effects, we conducted exploratory subgroup analyses by the following effect modifiers: (1) maternal age (<20 yrs, 20 to 29 yrs, or ≥30 yrs); (2) maternal education level (<8 yrs or ≥8 yrs); (3) parity (0 or ≥1); (4) maternal height (<150 cm or ≥150 cm); (5) pre-pregnancy or early-pregnancy BMI (underweight, normal-weight, overweight, or obesity); (6) anemia status at enrollment (no anemia: hemoglobin concentration ≥11.0 g/dL; mild anemia: hemoglobin concentration ≥10.0 and <11.0 g/dL; moderate to severe anemia: hemoglobin concentration <10.0 g/dL) [44]; (7) gestational age at enrollment (<20 weeks or ≥20 weeks); and (8) adherence to the assigned regimen (<90% or ≥90%). The cutoffs for these potential effect modifiers were pre-specified in accordance with previous studies [4,21,22]. We conducted stratified analyses within each study to obtain study- and stratum-specific estimates, which were then pooled using the meta-analytical approach to obtain the stratum-specific pooled estimates.

We conducted stratified analyses by study characteristics, including (1) geographic area (sub-Saharan Africa, South Asia, Latin America, and the Caribbean, and the Middle East and North Africa); (2) energy content of the supplementation (250 - <500 kcal/d, or 500 - <1,000); (3) forms of the BEP supplement (food rations or lipid-based supplement); and (4) targeting strategies of the BEP supplement (targeted or untargeted): targeted studies were defined as the studies in which the BEP supplements were provided to participants in the intervention arm under specific criteria (e.g., low BMI or low GWG), and untargeted studies were the studies in which the BEP supplements were universally provided to all participants allocated to the intervention arm.

We conducted random-effects meta-regression to explore potential sources of heterogeneity across the included studies. The study-level characteristics assessed in meta-regression included geographic area (Latin America and the Caribbean, Middle East and North Africa, South Asia, Sub-Saharan Africa), energy content of BEP supplements (per 100 kcal/day increment), forms of BEP supplements (food ration versus lipid-based), delivery strategies (targeted versus untargeted supplementation), and mean pre-pregnancy or early-pregnancy body mass index (per 1 kg/m^2^ increment). The outcomes for the meta-regression included GWG percent adequacy at the last weight measurement, estimated total GWG at delivery, and the risks of severely inadequate GWG, inadequate GWG, and excessive GWG. For each characteristic, we calculated the mean difference or RR with 95% CIs. We also reported the percentage of total between-study variance explained by each variable.

We conducted the study-specific analyses using SAS 9.4 (SAS Institute Inc., Cary, North Carolina). We used the PROC GENMOD function in SAS to conduct the linear, log-binomial, and modified Poisson models. We conducted the meta-analyses and meta-regression using Comprehensive Meta-Analysis Software Version 4 (Biostat Inc., Englewood, New Jersey, United States of America) [45]. The DerSimonian and Laird method was used for the meta-analyses. Statistical analyses were conducted using a two-sided *α* level of 0.05. We used a frequentist approach due to the objective of synthesizing empirical evidence without incorporating external prior information on the effects of prenatal BEP on GWG, which remained limited.

### Assessment of risk of bias

For individually randomized RCTs, we assessed the risk of bias (ROB) using the ROB-2 tool [46]. For cluster RCTs, we used the ROB-2 tool for cluster-randomized studies [47].

## Results

Eleven studies were included in the pooled analysis (**Table 1**) [23–33], including 2 studies conducted in Burkina Faso [24,31], 2 in Nepal [26,33], 2 in Pakistan [29,32], 1 in The Gambia [25], 1 in India [30], 1 in Iran [23], 1 in Mexico [28], and 1 multicountry study with data from Guatemala, India, and Pakistan [27]. Three of the included studies [26,28,29] were cluster RCTs and the other 8 used individual randomization. Six studies [24,25,27,31–33] used lipid-based supplements as BEP, and the other 5 [23,26,28–30] used BEP in the form of food rations. The daily energy content of the BEP supplements ranged from 250 kcal to 970 kcal per day. The comparison groups varied across studies and included standard of care, iron, and folic acid supplements, multiple micronutrient supplements (MMS), micronutrient powder, and antenatal counseling. Two studies, the Women First Study [27] and the WINGS Study [30], provided prenatal BEP supplements in the intervention arm using a targeted approach toward pregnant women with a low BMI or a low GWG. Specifically, in the Women First Study, an SQ-LNS of 118 kcal and 2.6 g protein per day was provided to all participants in the intervention arm, and those with BMI <20 at any time or inadequate GWG in the second or third trimesters received additional unfortified lipid-based supplement providing additional 300 kcal and 11 g protein per day [27]. In the WINGS study, locally prepared snacks (210 kcal and 2 g protein per day in the second trimester, and 400 kcal and 21 g protein per day in the third trimester) were provided for pregnant women with BMI <25, and additional hot cooked meals (500 kcal and 20 g protein per day) were provided for those with BMI <18.5 or inadequate GWG [30]. In the other 9 studies, BEP supplements were provided untargeted so that all participants randomly allocated to the intervention arm received BEP supplements.

**Table 2** shows the distribution of gestational weight gain outcomes by study and in the overall analytical sample. The mean GWG percent adequacy at the last weight measurement ranged from 59.4% to 127.5% across the included studies, with an overall mean of 75.2% (78.1% in the BEP arm and 72.8% in the control arm). The mean estimated total GWG at delivery ranged from 7.5 kg to 12.2 kg across studies, with an overall mean of 9.1 kg (9.4 kg in the BEP arm and 8.9 kg in the control arm). The percentage of participants with severely inadequate GWG ranged from 7.1% to 65.6% across studies, with an overall percentage of 45.8% (44.3% in the BEP arm and 47.1% in the control arm). The percentage of participants with inadequate GWG ranged from 23.7% to 85.5% across studies, with an overall percentage of 71.9% (68.3% in the BEP arm and 74.9% in the control arm). The percentage of participants with excessive GWG ranged from 2.8% to 43.6% across studies, with an overall percentage of 9.8% (11.2% in the BEP arm and 8.6% in the control arm).

**Table 2:** Distribution of gestational weight gain outcomes by study and in overall analytical sample^1^.

Study	Number of participants			GWG percent adequacy at last weight measurement^2^			Estimated total GWG at delivery^3^, kg			Severely inadequate GWG^4^			Inadequate GWG^4^			Excessive GWG^4^		
	Total	BEP	Control	Total	BEP	Control	Total	BEP	Control	Total	BEP	Control	Total	BEP	Control	Total	BEP	Control
Kaseb, 2002	52	28	24	127.5 (60.5)	131.0 (58.1)	123.5 (64.2)	NA^5^	NA^5^	NA^5^	17.3	14.3	20.8	30.8	28.6	33.3	38.5	39.3	37.5
Huybregts, 2009	1,134	565	569	59.4 (35.9)	62.4 (38.1)	56.5 (33.3)	7.5 (4.5)	7.8 (4.7)	7.2 (4.2)	65.6	63.4	67.8	85.5	83.2	87.9	2.8	2.8	2.8
Moore, 2012	821	405	416	62.1 (52.1)	63.6 (49.0)	60.6 (55.0)	8.1 (5.7)	8.2 (5.4)	8.1 (5.9)	57.5	57.5	57.5	75.5	75.3	75.7	8.0	9.1	7.0
Saville, 2018	2,555	743	1,812	75.4 (25.4)	76.4 (27.9)	75.0 (24.4)	9.7 (2.8)	9.8 (3.1)	9.7 (2.7)	37.2	37.3	37.1	84.8	83.6	85.3	3.4	4.4	2.9
Hambidge, 2019	1,308	687	621	69.3 (49.6)	72.0 (47.0)	66.3 (52.3)	8.3 (5.4)	8.5 (5.1)	8.0 (5.6)	52.5	49.2	56.2	72.9	70.7	75.4	10.4	9.8	11.1
Neufeld, 2019	422	153	269	126.4 (52.1)	128.3 (56.5)	123.5 (64.2)	12.2 (3.8)	12.1 (3.9)	12.2 (3.7)	7.1	6.5	7.4	23.7	22.9	24.2	43.6	44.4	43.1
Khan, 2021	1,161	592	569	74.0 (37.6)	75.9 (41.8)	72.1 (32.5)	9.6 (3.8)	9.7 (4.2)	9.4 (3.4)	48.4	46.5	50.4	75.8	73.1	78.6	5.1	5.6	4.6
Taneja, 2022	2,089	1,042	1,047	83.0 (51.0)	92.1 (49.2)	73.9 (51.1)	9.5 (5.4)	10.6 (5.3)	8.5 (5.3)	39.9	31.9	47.9	59.5	52.2	66.7	17.0	21.7	12.4
de Kok, 2022	1,723	837	886	72.7 (42.3)	75.0 (42.2)	70.5 (42.2)	8.4 (4.5)	8.7 (4.5)	8.2 (4.5)	49.6	47.9	51.1	69.8	67.5	72.0	8.5	9.2	7.8
Muhammad, 2022	567	282	285	78.8 (59.8)	82.1 (64.1)	75.5 (55.1)	9.1 (6.6)	9.6 (7.5)	8.7 (5.6)	48.3	47.2	49.5	68.4	67.4	69.5	12.5	12.1	13.0
Erchick, 2023	717	359	358	74.5 (42.0)	76.7 (41.0)	72.3 (42.9)	9.6 (5.2)	9.9 (5.1)	9.4 (5.4)	46.3	44.6	48.0	67.0	64.4	69.6	10.6	10.6	10.6
Overall	12,549	5,693	6,856	75.2 (44.8)	78.1 (46.3)	72.8 (43.4)	9.1 (4.7)	9.4 (5.0)	8.9 (4.5)	45.8	44.3	47.1	71.9	68.3	74.9	9.8	11.2	8.6

^1^ BEP, balanced energy and protein; GWG, gestational weight gain; IOM, Institute of Medicine.

^2^ Calculated by dividing the observed GWG at the last weight measure during pregnancy by the recommended GWG according to the Institute of Medicine guidelines, multiplied by 100. Values shown are means (standard deviations).

^3^ Calculated by multiplying the GWG percent adequacy at the last available weight measurement by the IOM-recommended GWG at delivery, which was computed based on individual-specific gestational age at delivery and body mass index category. Values shown are means (standard deviations).

^4^ Severely inadequate, inadequate, and excessive were defined as <70%, <90%, and >125% of GWG percent adequacy at the last weight measurement, respectively. Values shown are percentages.

^5^ Estimated total GWG at delivery cannot be calculated for Kaseb, 2002, due to the lack of data on gestational age at delivery, which is necessary for the computation of estimated total GWG at delivery.

**Fig 1:**
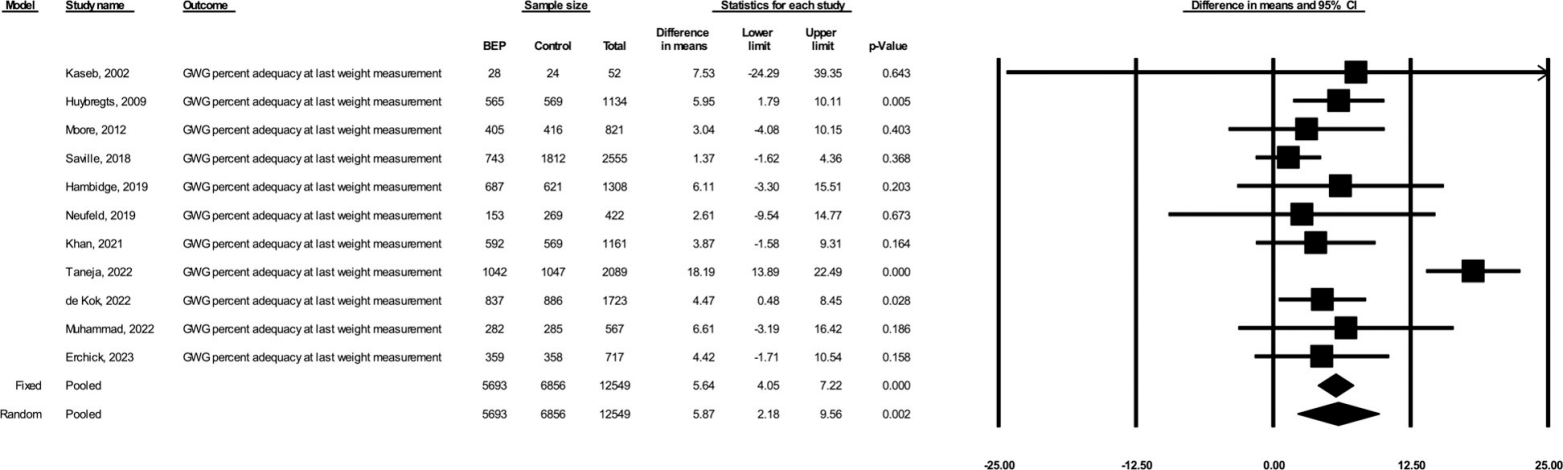
Forest plot for the effect of prenatal BEP supplements on GWG percent adequacy at the last gestational weight measurement. BEP, balanced energy and protein; CI, confidence interval; GWG, gestational weight gain.

**Fig 2:**
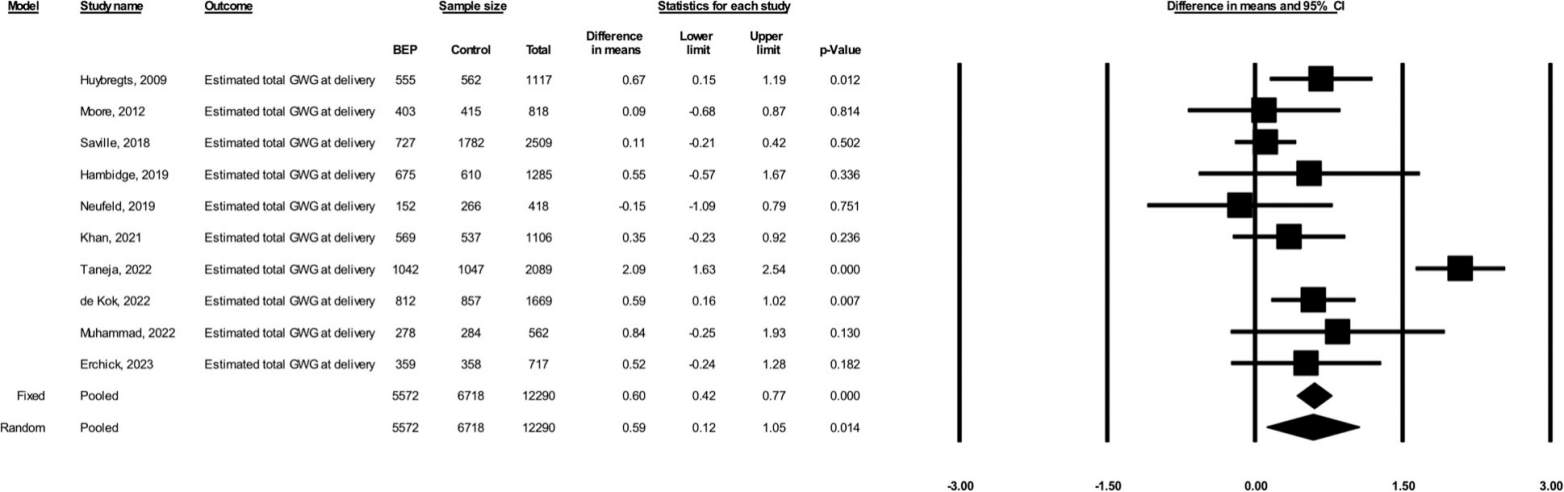
Forest plot for the effect of prenatal BEP supplements on the estimated total GWG at delivery (kilograms). BEP, balanced energy and protein; CI, confidence interval; GWG, gestational weight gain.

**Fig 3:**
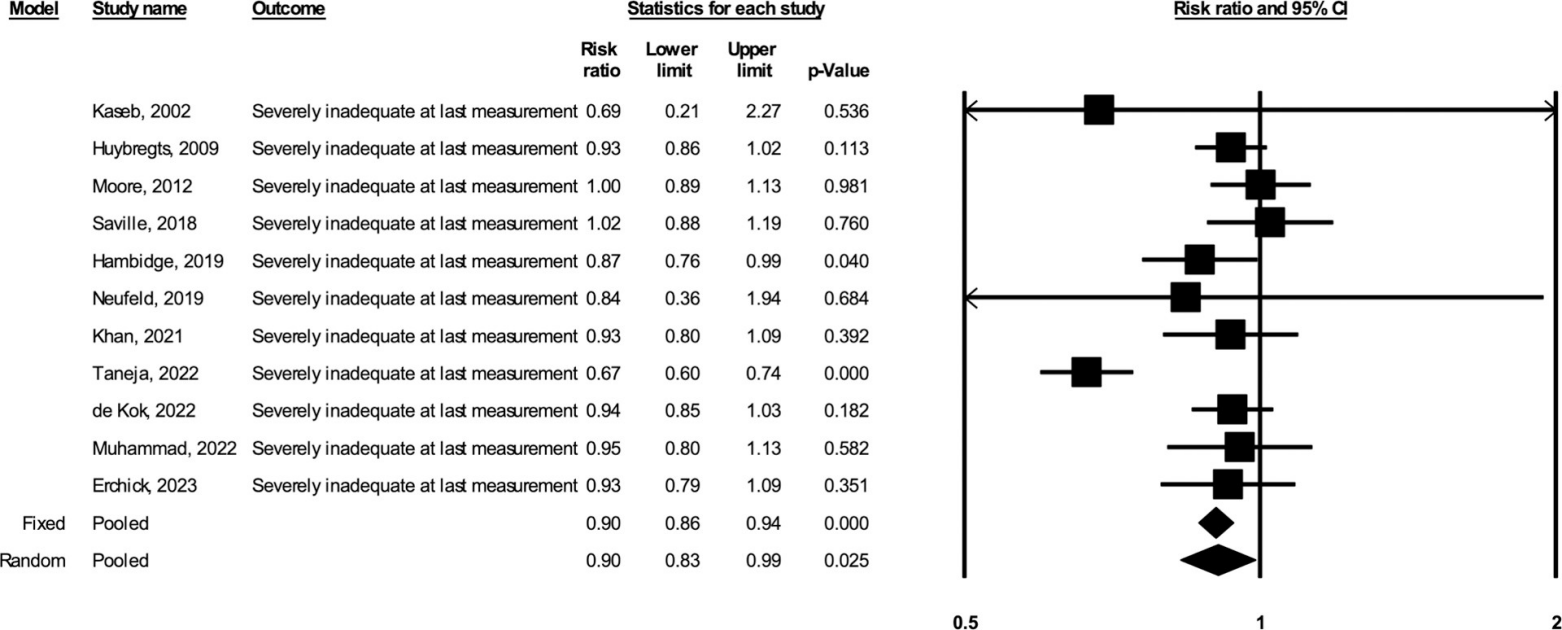
Forest plot for the effect of prenatal BEP supplements on severely inadequate GWG at the last gestational weight measurement. BEP, balanced energy and protein; CI, confidence interval; GWG, gestational weight gain.

**Fig 4:**
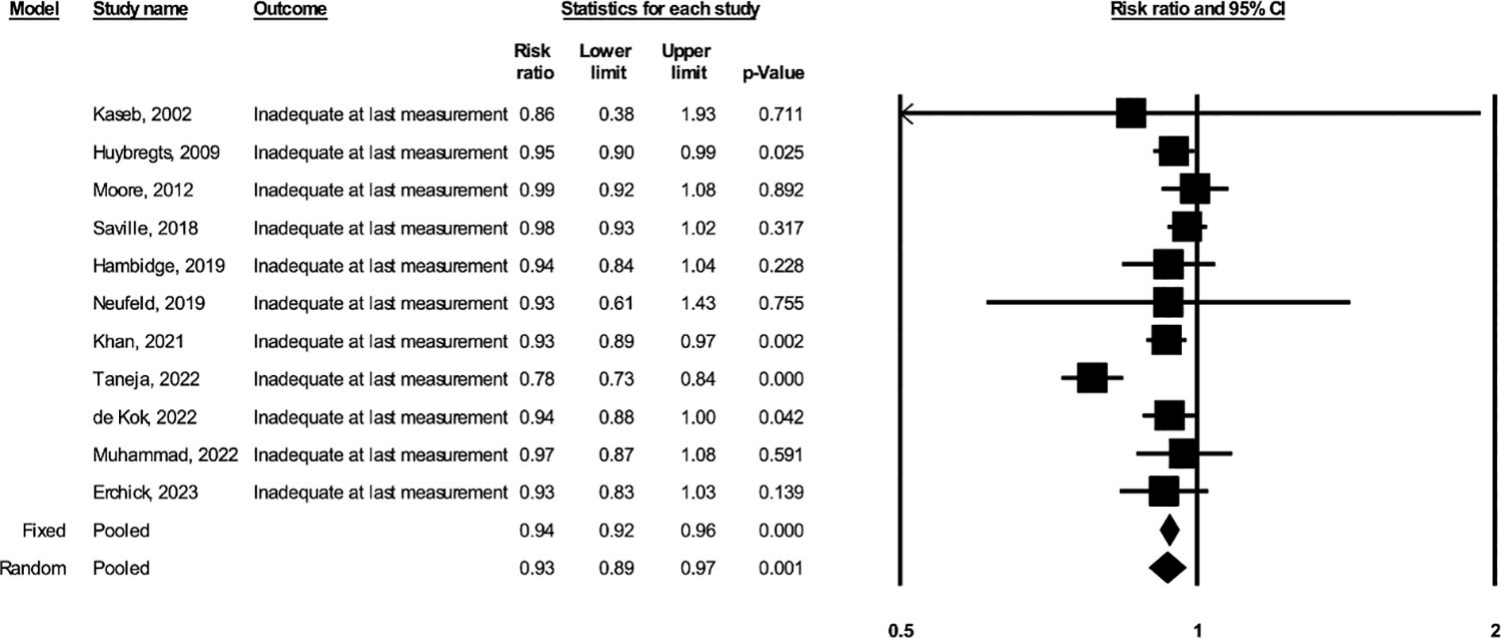
Forest plot for the effect of prenatal BEP supplements on inadequate GWG at the last gestational weight measurement. BEP, balanced energy and protein; CI, confidence interval; GWG, gestational weight gain.

**Fig 5:**
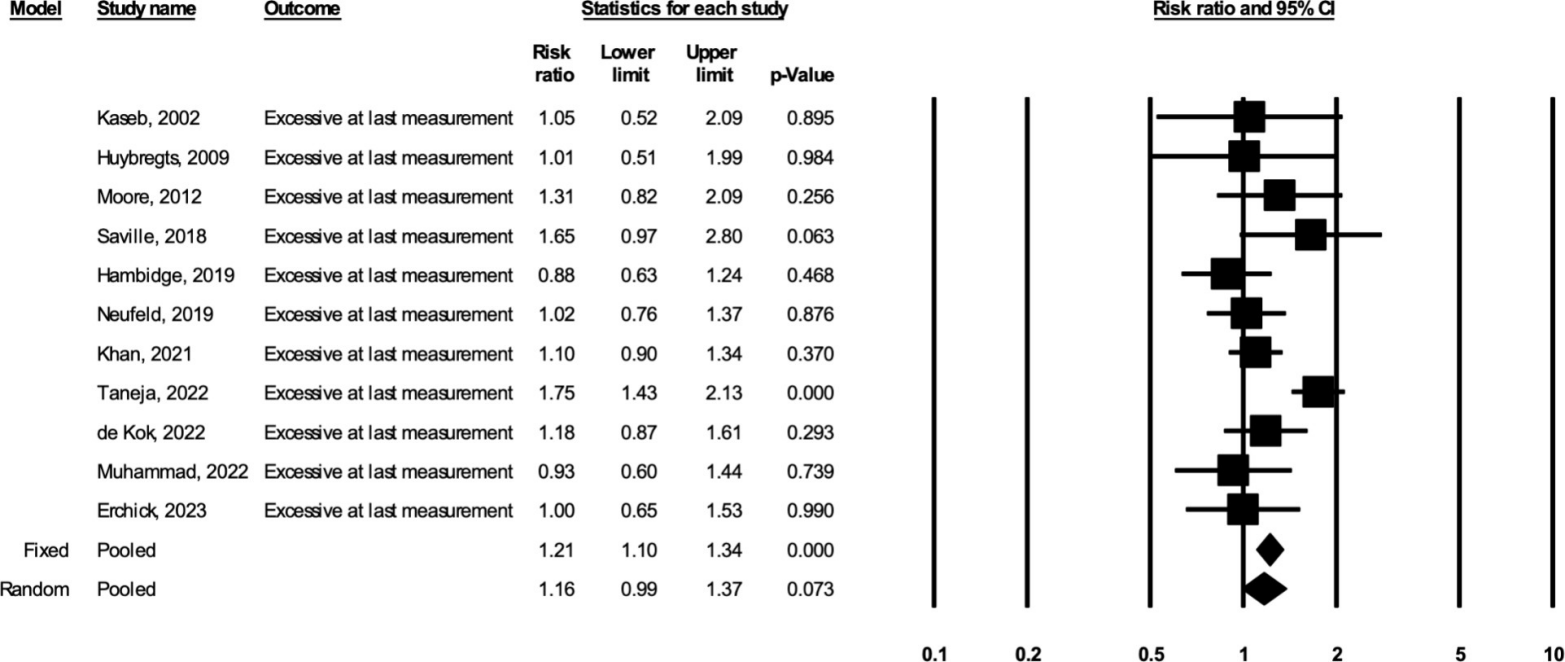
Forest plot for the effect of prenatal BEP supplements on excessive GWG at the last gestational weight measurement. BEP, balanced energy and protein; CI, confidence interval; GWG, gestational weight gain.

Based on the random-effects models including 11 studies (total *N* = 12,549, including 5,693 participants in the BEP arm and 6,856 participants in the comparison arm), prenatal BEP led to a 5.87% greater GWG percent adequacy at the last weight measurement (95% CI: 2.18, 9.56; *p* = 0.002; I^2^ = 76%) compared to comparison (**Fig 1**). Based on the random-effects models including 10 studies (total *N* = 12,290, including 5,572 in the BEP arm and 6,718 in the comparison arm), prenatal BEP resulted in a 0.59 kg greater estimated total GWG at delivery (95% CI: 0.12, 1.05; *p* = 0.014; I^2^ = 84%) (**Fig 2**). Prenatal BEP resulted in a 10% lower risk of having severely inadequate GWG (RR: 0.90; 95% CI: 0.83, 0.99; *p* = 0.025; I^2^ = 74%) (**Fig 3**) and a 7% lower risk of having inadequate GWG (RR: 0.93; 95% CI: 0.89, 0.97; *p* = 0.001; I^2^ = 66%) (**Fig 4**). In the random-effects model, prenatal BEP did not have a significant effect on the risk of having excessive GWG (RR: 1.16; 95% CI: 0.99, 1.37; *p* = 0.073; I^2^ = 56%) (**Fig 5**) at the last weight measurement compared to comparison. In the analysis for the weekly rate of GWG within the second and third trimesters, prenatal BEP did not have a significant effect on inadequate weekly GWG within the second trimester (**S2 Fig**) but led to a higher risk of excessive weekly GWG within the second trimester (RR: 1.18; 95% CI: 1.02, 1.37; *p* = 0.03; I^2^ = 0%; **S3 Fig**). Prenatal BEP did not have significant effects on the risks of inadequate or excessive weekly GWG within the third trimester (**S4** and **S5 Figs**). In the sensitivity analyses adjusting for covariates including maternal age, maternal years of education, parity, gestational age at enrollment, maternal height, pre-pregnancy or early-pregnancy BMI, and hemoglobin concentration at enrollment, the study-specific estimates were similar to those from the primary analyses (**S3 Table**). Similar results were also obtained when multiple imputation was used for missing covariate data (**S4 Table**).

In subgroup analyses by individual-level maternal characteristics (**Table 3**), the effect of prenatal BEP on GWG was greater among participants with normal weight before pregnancy or during early pregnancy than participants with underweight, and even greater among participants with overweight or obesity (*P* for interaction: 0.003 for GWG percent adequacy at least measurement; 0.002 for severely inadequate GWG; and 0.016 for inadequate GWG). The effect of prenatal BEP on the risk of excessive GWG was stronger among participants enrolled before 20 weeks of gestation (RR: 1.39; 95% CI: 1.16, 1.67) compared to those enrolled at 20 weeks or later (RR: 0.95; 95% CI: 0.70, 1.30; *P* for interaction: 0.04). Prenatal BEP was associated with a lower risk of inadequate GWG among participants with no anemia (RR: 0.89; 95% CI: 0.83, 0.95) or mild anemia (RR: 0.92; 95% CI: 0.86, 0.99), but not among those with moderate to severe anemia (RR: 0.98; 95% CI: 0.92, 1.05). Prenatal BEP was associated with an increased risk of excessive GWG among participants with no anemia (RR: 1.40; 95% CI: 1.11, 1.78) or mild anemia (RR: 1.61; 95% CI: 1.17, 2.21), but not among those with moderate to severe anemia (RR: 0.82; 95% CI: 0.63, 1.07). There was no indication of effect modification by maternal age, parity, maternal height, or adherence to the assigned regimen.

**Table 3:** The effect of prenatal BEP supplements on GWG by maternal characteristics^1^.

	GWG percent adequacy at last weight measurement^2^, %			Estimated total GWG at delivery^3^, kg			Severely inadequate GWG^4^			Inadequate GWG^4^			Excessive GWG^4^		
Subgroup	Number of studies	WMD (95% CI)^5^	*P* for interaction	Number of studies	WMD (95% CI)^5^	*P* for interaction	Number of studies	Pooled RR (95% CI)^6^	*P* for interaction	Number of studies	Pooled RR (95% CI)^6^	*P* for interaction	Number of studies	Pooled RR (95% CI)^6^	*P* for interaction
Maternal age, years															
<20	10	5.73 (−0.56, 12.03)	0.98	10	0.66 (−0.10, 1.42)	0.83	10	0.91 (0.78, 1.06)	0.80	10	0.92 (0.85, 1.00)	0.75	7	1.43 (1.03, 1.96)	0.52
20–29	10	6.34 (1.32, 11.36)		10	0.68 (0.06, 1.31)		10	0.89 (0.78, 1.00)		10	0.93 (0.88, 0.99)		7	1.17 (0.95, 1.45)	
≥30	10	5.56 (−0.32, 11.43)		10	0.41 (−0.28, 1.10)		10	0.94 (0.82, 1.09)		10	0.96 (0.89, 1.03)		7	1.13 (0.84, 1.51)	
Maternal education, years															
<8	6	4.66 (0.65, 8.67)	0.94	6	0.49 (0.00, 0.97)	0.38	6	0.93 (0.88, 0.99)	0.053	6	0.95 (0.91, 0.99)	0.70	5	1.23 (0.89, 1.71)	0.65
≥8	6	4.39 (−1.59, 10.38)		6	0.83 (0.25, 1.41)		6	0.88 (0.86, 0.89)		6	0.97 (0.90, 1.04)		5	1.09 (0.70, 1.69)	
Parity															
0	7	4.17 (0.45, 7.89)	0.94	7	0.45 (−0.05, 0.94)	0.87	7	0.90 (0.82, 0.99)	0.44	7	0.93 (0.89, 0.97)	0.28	5	1.17 (0.76, 1.79)	0.63
≥1	7	4.00 (1.82, 6.17)		7	0.40 (0.11, 0.69)		7	0.94 (0.89, 0.99)		7	0.96 (0.93, 0.99)		5	1.04 (0.92, 1.18)	
Maternal height, cm															
<150	11	6.83 (1.90, 11.76)	0.67	10	0.68 (0.09, 1.27)	0.72	11	0.89 (0.80, 1.00)	0.86	10	0.95 (0.90, 1.01)	0.39	8	1.03 (0.79, 1.35)	0.50
≥150	11	5.44 (1.33, 9.56)		10	0.54 (0.03, 1.05)		11	0.90 (0.83, 0.99)		10	0.92 (0.88, 0.97)		8	1.18 (0.91, 1.51)	
Pre-pregnancy or early-pregnancy BMI, kg/m^2^															
Underweight	10	1.37 (−1.90, 4.65)	0.003	10	0.26 (−0.21, 0.73)	0.065	9	1.00 (0.91, 1.09)	0.002	8	0.98 (0.93, 1.04)	0.016	8	0.76 (0.47, 1.21)	0.19
Normal weight	10	4.79 (1.65, 7.93)		10	0.60 (0.18, 1.02)		9	0.89 (0.82, 0.96)		8	0.93 (0.89, 0.98)		8	1.25 (0.96, 1.61)	
Overweight or obesity	10	15.69 (7.90, 23.47)		10	1.22 (0.57, 1.88)		9	0.75 (0.65, 0.86)		8	0.81 (0.72, 0.92)		8	1.14 (0.87, 1.48)	
Anemia at enrollment															
No anemia	7	9.34 (3.46, 15.22)	0.36	7	0.91 (0.28, 1.53)	0.59	6	0.88 (0.77, 1.00)	0.89	6	0.89 (0.83, 0.95)	0.093	6	1.40 (1.11, 1.78)	0.002
Mild anemia	7	7.93 (2.25, 13.61)		7	0.60 (−0.04, 1.23)		6	0.87 (0.76, 1.00)		6	0.92 (0.86, 0.99)		6	1.61 (1.17, 2.21)	
Moderate to severe anemia	7	3.64 (−2.01, 9.28)		7	0.44 (−0.20, 1.09)		6	0.91 (0.80, 1.04)		6	0.98 (0.92, 1.05)		6	0.82 (0.63, 1.07)	
Gestational age at enrollment, weeks															
<20	9	6.43 (2.40, 10.46)	0.38	9	0.70 (0.24, 1.16)	0.18	8	0.91 (0.82, 1.01)	0.74	8	0.92 (0.89, 0.97)	0.22	5	1.39 (1.16, 1.67)	0.04
≥20	9	3.50 (−1.63, 8.63)		9	0.20 (−0.39, 0.78)		8	0.94 (0.79, 1.13)		8	0.97 (0.91, 1.04)		5	0.95 (0.70, 1.30)	
Adherence to assigned regimen, %															
<90	1	4.81 (−0.32, 9.93)	0.36	1	0.48 (−0.14, 1.10)	0.75	1	0.94 (0.85, 1.04)	1.00	1	0.96 (0.76, 1.21)	0.79	1	1.30 (0.38, 4.40)	0.54
≥90	1	8.94 (1.63, 16.24)		1	1.17 (0.22, 2.13)		1	0.94 (0.81, 1.09)		1	0.92 (0.72, 1.17)		1	0.73 (0.19, 2.90)	

^1^ BEP, balanced energy and protein; BMI, body mass index; CI, confidence interval; GWG, gestational weight gain; IOM, Institute of Medicine; RR, risk ratio; WMD, weighted mean difference.

^2^ Calculated by dividing the observed GWG at the last weight measure during pregnancy by the recommended GWG according to the Institute of Medicine guidelines, multiplied by 100.

^3^ Calculated by multiplying the GWG percent adequacy at the last available weight measurement by the IOM-recommended GWG at delivery, which was computed based on individual-specific gestational age at delivery and body mass index category.

^4^ Severely inadequate, inadequate, and excessive were defined as <70%, <90%, and >125%, respectively.

^5^ Values are weighted mean differences and 95% CIs from random-effects meta-analytical models comparing BEP with comparison.

^6^ Values are pooled risk ratios and 95% CIs from random-effects meta-analytical models comparing BEP with comparison.

In subgroup analyses by study-level characteristics (**Table 4**), among studies using BEP supplements of 500 to less than 1,000 kcal/d, BEP increased the risk of excessive GWG (RR: 1.32; 95% CI: 1.08, 1.62), whereas this effect was not observed in studies using supplements of 250 to less than 500 kcal (RR: 1.02; 95% CI: 0.83, 1.26). The effects of prenatal BEP on GWG outcomes were stronger in studies with a targeted approach to BEP provision. Specifically, prenatal BEP had a greater effect on GWG percent adequacy at the last weight measurement (targeted studies: WMD: 16.02% [95% CI: 12.00, 20.03]; untargeted studies: WMD: 3.61% [95% CI: 1.81, 5.42]; *P* for interaction: <0.001), estimated total GWG at delivery (targeted studies: WMD: 1.73 kg [95% CI: 1.14, 2.32]; untargeted studies: WMD: 0.37 kg [95% CI: 0.09, 0.65]; *P* for interaction: <0.001), severely inadequate GWG (targeted studies: RR: 0.74 [95% CI: 0.67, 0.82]; untargeted studies: RR: 0.95 [95% CI: 0.90, 1.01]; *P* for interaction: <0.001), and inadequate GWG (targeted studies: RR: 0.83 [95% CI: 0.78, 0.89]; untargeted studies: RR: 0.95 [95% CI: 0.93, 0.98]; *P* for interaction: <0.001).

**Table 4:** The effects of prenatal BEP supplements on GWG by study setting and nutritional composition of the supplements^1^.

	GWG percent adequacy at last weight measurement^2^, %			Estimated total GWG at delivery^3^, kg			Severely inadequate GWG^4^			Inadequate GWG^4^			Excessive GWG^4^		
Subgroup	Number of studies	WMD (95% CI)^5^	*P* for interaction	Number of studies	WMD (95% CI)^5^	*P* for interaction	Number of studies	Pooled RR (95% CI)^6^	*P* for interaction	Number of studies	Pooled RR (95% CI)^6^	*P* for interaction	Number of studies	Pooled RR (95% CI)^6^	*P* for interaction
Geographic area^7^															
Sub-Saharan Africa	3	4.58 (−2.72, 11.87)	0.90	3	0.47 (−0.47, 1.41)	0.95	3	0.96 (0.83, 1.10)	0.52	3	0.96 (0.89, 1.03)	0.47	3	1.18 (0.80, 1.74)	0.99
South Asia	7	5.89 (0.86, 10.92)		7	0.63 (−0.01, 1.28)		7	0.89 (0.80, 0.99)		7	0.93 (0.88, 0.99)		7	1.13 (0.89, 1.44)	
Latin America and the Caribbean	2	9.74 (−1.28, 20.75)		2	0.67 (−0.58, 1.93)		2	0.76 (0.58, 1.00)		2	0.83 (0.71, 0.97)		2	1.07 (0.69, 1.65)	
Middle East and North Africa	1	7.53 (−26.31, 41.38)		0	NA		1	0.69 (0.20, 2.32)		1	0.86 (0.38, 1.95)		1	1.05 (0.45, 2.44)	
Energy content of BEP supplements, kcal/d															
250 - < 500	6	4.93 (−0.81, 10.67)	0.66	5	0.46 (−0.27, 1.19)	0.64	6	0.91 (0.80, 1.04)	0.87	6	0.94 (0.87, 1.00)	0.82	6	1.02 (0.83, 1.26)	0.078
500 - < 1,000	5	6.73 (1.14, 12.31)		5	0.70 (−0.01, 1.41)		5	0.90 (0.79, 1.02)		5	0.93 (0.87, 0.98)		5	1.32 (1.08, 1.62)	
Forms of BEP															
Food ration	5	7.01 (0.82, 13.20)	0.63	4	0.64 (−0.14, 1.42)	0.85	5	0.84 (0.73, 0.97)	0.22	5	0.90 (0.84, 0.97)	0.23	5	1.29 (1.04, 1.60)	0.17
Lipid-based supplement	6	5.02 (−0.25, 10.28)		6	0.54 (−0.13, 1.21)		6	0.94 (0.85, 1.03)		6	0.95 (0.90, 1.01)		6	1.04 (0.83, 1.30)	
Delivery strategies of BEP															
Targeted	2	16.02 (12.00, 20.03)	< 0.001	2	1.73 (1.14, 2.32)	< 0.001	2	0.74 (0.67, 0.82)	< 0.001	2	0.83 (0.78, 0.89)	< 0.001	2	1.33 (1.00, 1.78)	0.29
Untargeted	9	3.61 (1.81, 5.42)		8	0.37 (0.09, 0.65)		9	0.95 (0.90, 1.01)		9	0.95 (0.93, 0.98)		9	1.11 (0.94, 1.32)	

^1^ BEP, balanced energy and protein; CI, confidence interval; GWG, gestational weight gain; IOM, Institute of Medicine; RR, risk ratio; WMD, weighted mean difference.

^2^ Calculated by dividing the observed GWG at the last weight measure during pregnancy by the recommended GWG according to the Institute of Medicine guidelines, multiplied by 100.

^3^ Calculated by multiplying the GWG percent adequacy at the last available weight measurement by the IOM-recommended GWG at delivery, which was computed based on individual-specific gestational age at delivery and body mass index category.

^4^ Severely inadequate, inadequate, and excessive were defined as <70%, <90%, and >125%, respectively.

^5^ Values are weighted mean differences and 95% CIs from random-effects meta-analytical models comparing BEP with comparison.

^6^ Values are pooled risk ratios and 95% CIs from random-effects meta-analytical models comparing BEP with comparison.

^7^ For the Women First Study, a multicountry study conducted in Guatemala, India, and Pakistan, the country-specific estimates were calculated and used in the corresponding category of geographic area.

In meta-regression that further explored the sources of heterogeneity across studies (**S5 Table**), geographic area did not explain any of the between-study heterogeneity across outcomes. A higher energy content in the BEP supplements was associated with a higher risk of excessive GWG (for each 100 kcal/day increment of energy content: RR: 1.08; 95% CI: 1.03, 1.13; *P* = 0.008); energy content of the BEP supplements explained 96% of the between-study variance in the effect of BEP on excessive GWG. Energy content explained 49%, 42%, 31%, and 26% of the between-study variance in the effect of BEP for GWG percent adequacy, estimated total GWG at delivery, severely inadequate GWG, and inadequate GWG, respectively. The form of BEP supplementation (food ration versus lipid-based supplement) did not explain a sizable portion of the heterogeneity, except for severely inadequate GWG, where it explained 24% of the variance, and excessive GWG, where it explained 21% of the variance (though neither were statistically significant). Regarding the delivery strategy of BEP supplementation, the targeted delivery strategy explained a large portion of the heterogeneity, accounting for 99%, 86%, 90%, 88%, and 32% of the between-study variance for GWG percent adequacy, total GWG at delivery, severely inadequate GWG, inadequate GWG, and excessive GWG, respectively. The mean pre-pregnancy or early-pregnancy BMI was not significantly associated with any of the outcomes, although it explained 30% of the between-study variance in GWG percent adequacy and 36% of the between-study variance in the risk of severely inadequate GWG. Of the 11 studies included in this analysis, 10 were assessed to have a low risk of bias (**S6 Table**), and one had a high risk of bias due to the potential bias arising from the randomization process [23].

## Discussion

In this individual participant data meta-analysis of RCTs conducted in LMICs, we find that prenatal BEP supplements increase GWG and reduce the risk of gaining inadequate weight during pregnancy. The effects of prenatal BEP on GWG outcomes may be stronger in studies with a targeted approach, where BEP supplements were provided to participants in the intervention arm under specific criteria such as low body mass index or low GWG, compared to studies with an untargeted approach, where BEP supplements were provided to all participants allocated to the intervention arm.

GWG is a powerful indicator of maternal and fetal nutrition and is increasingly recognized as a target for antenatal monitoring and interventions. Few studies have examined the effect of prenatal BEP supplements on GWG. We find that prenatal supplements increase total GWG at delivery by 0.59 kg. This finding is congruent with a prior meta-analysis of 10 studies and 2,571 participants showing that prenatal BEP increased the weekly rate of GWG by 20.74 grams/week [16], which, assuming term deliveries at 40 weeks, would roughly correspond to a difference of 0.54 kg in total GWG during the second and third trimesters. Our findings add to the limited body of evidence supporting the effect of prenatal BEP in increasing GWG. In LMICs with a higher prevalence of undernutrition, an increase of the total GWG by 0.59 kg holds clinical significance, as it corresponds to a 7% lower risk of inadequate GWG and a 10% lower risk of severely inadequate GWG. Future efforts are needed to examine to what extent the beneficial effect of prenatal BEP supplements on birth outcomes may be mediated by the preventive effect against inadequate GWG.

This study shows that prenatal BEP supplements providing considerable energy content may have stronger effects on GWG than other commonly used prenatal supplements, such as MMS and SQ-LNS. In another meta-analysis using individual participant data, we showed that prenatal MMS increased GWG percent adequacy by 0.86% and the estimated total GWG at delivery by 0.21 kg, and reduced the risk of severely inadequate GWG by 2.9% and the risk of inadequate GWG by 1.4% [21]. The same meta-analysis found no significant effect of SQ-LNS on GWG [21]. In contrast, in the present analysis, we find that prenatal BEP increases GWG percent adequacy by 5.87% and total GWG at delivery by 0.59 kg and reduces the risk of severely inadequate GWG by 10% and the risk of inadequate GWG by 7%. All BEP supplements examined in this analysis contained 250 kcal or greater calories per day, compared to zero energy from MMS and less than 120 kcal per day in SQ-LNS. Therefore, the greater effect of the BEP supplements in this analysis compared to MMS and SQ-LNS may be partially explained by the greater energy content.

We found that the positive effect of prenatal BEP on GWG was greater among participants with normal weight, overweight, or obesity than participants with underweight before pregnancy. Women with underweight before pregnancy may have more pronounced macro- and micronutrient deficiencies that are not fully addressed by the BEP supplements provided, which could limit the effect of BEP on GWG. In contrast, normal weight or overweight/obese women might have more adequate baseline nutrition, allowing them to respond more effectively to the additional energy, protein, and micronutrients. Another explanation may be that the GWG target based on the IOM recommendation is greater for women with underweight, thus making it more challenging for underweight women to achieve a greater GWG adequacy [1]. We also found that the effect of BEP on GWG was stronger among women enrolled prior to 20 weeks of gestation. While starting the BEP supplementation earlier enables more time for the supplements to take effect to prevent inadequate GWG, the risk of excessive GWG from a longer duration of supplementation cannot be ruled out. With BEP supplements, careful monitoring of weight gain may be warranted to prevent excessive GWG, especially when the supplements start early in pregnancy.

Despite the accumulating evidence on the efficacy of prenatal BEP in preventing adverse birth outcomes, programmatic gaps remain in the cost-effective delivery strategies of BEP in low-resource settings. The antenatal guidelines by the WHO recommend that BEP supplements be provided in populations with a high prevalence of undernutrition, defined as a geographical area with a prevalence of underweight of 20% or greater [48]. This universal delivery strategy may provide BEP supplements to low-risk pregnant women and may also miss vulnerable pregnant women who may benefit substantially from the intervention but do not reside in a high-risk area. It has been recognized that targeting strategies based on individual nutritional status may be more impactful and cost-effective than the population-based approach [49]. Some specific targeting strategies based on individual nutritional status include targeting by BMI, mid-upper arm circumference, GWG, or a combination of different anthropometric measures [49]. Our analysis showed that the effect of prenatal BEP on increased GWG adequacy was stronger in studies where BEP supplements were provided based on individual nutritional status, such as underweight and inadequate GWG. Meta-regression also showed that the delivery strategy of BEP supplementation was an important determinant of the effect size of the supplements for most of the GWG outcomes examined. These findings provide support for targeted BEP supplementation. However, the apparently greater effects with targeting should be interpreted with caution, as most of the included trials were not specifically targeted for women with undernutrition, and only 2 studies used a targeted approach [27,30], with one of them [30] being a multicomponent trial that included interventions to reduce infection and inflammation. The composition of BEP supplements used in blanket supplementation programs might differ from what would be considered ideal for those with undernutrition, who may require higher amounts of macronutrients and more specific formulations to address nutritional deficiencies. The use of more optimal BEP formulations, particularly those providing medium or large quantities of macronutrients, may offer greater benefits in improving GWG among women with undernutrition. Efforts are underway to understand the effectiveness and cost-effectiveness of different strategies of BEP tailored to the nutritional needs of vulnerable pregnant women [50].

We did not find substantial evidence in the overall analysis to indicate that prenatal BEP supplements increase the risk of excessive GWG. In the subgroup analysis by the energy content of the supplements, we found that in studies that provided BEP supplements greater than 500 kcal of calories per day, the supplements may increase the risk of excessive GWG. This effect, however, was not seen in studies using supplements of 250 to less than 500 kcal. Meta-regression also shows that the energy content of the BEP supplements explained a large proportion of the heterogeneity in the effect size of BEP supplements on excessive GWG. These findings are consistent with the expert consultation at the Bill & Melinda Gates Foundation, which recommended that the energy content of BEP supplements should be between 250 and 500 kcal per daily serving [51]. Further, the GWG target is easier to achieve for women with overweight or obesity compared to those with underweight or normal weight. As a result, women with overweight and obesity may be more likely to reach excessive GWG when provided with an undue amount of BEP supplements [1]. The presence of considerable heterogeneity across most GWG outcomes underscores the need for context-specific strategies when designing and implementing BEP interventions. BEP supplements with energy content tailored to individual needs may maximize their benefits and minimize unfavorable effects, such as excessive GWG.

The strengths of this study include a large sample size, the inclusion of participants from 11 studies representing 8 LMICs from different world regions, and the use of individual participant data, the latter of which allowed for the calculation of GWG adequacy in a uniform approach. Our outcomes based on GWG percent adequacy were largely independent of gestational duration, thus reducing the biases inherent in some conventional measures of GWG, such as total GWG or average rate of GWG [20]. This study has several limitations. First, we could not include all BEP studies we identified. The main reasons for the lack of inclusion were the lack of response from the corresponding authors and the inability to contribute individual-level data. Some of the non-included studies have examined the effects of BEP and GWG and found an effect of BEP on increased total GWG [52] or weekly rate of GWG [53,54]. Second, the numerous subgroup analyses based on individual-level maternal characteristics were exploratory and likely underpowered. These subgroup analyses also depended on data availability on the potential effect modifiers, which were not always available across all studies. Additional effect modifiers not included in this analysis, such as physical activity and habitual dietary intake, also warrant investigation. Further, we did not adjust for multiple comparisons resulting from the numerous potential effect modifiers, which may increase the probability of type I error. Future studies specifically powered to examine various effect modifiers will be useful in confirming the findings from our subgroup analyses. Third, GWG adequacy in this work was defined using the IOM recommendations, which were developed using data primarily from the United States and thus might not represent the ideal GWG recommendations across diverse LMIC settings. For example, there is insufficient evidence regarding the utility of IOM recommendations among Indian and other Asian women [55]. However, the IOM recommendations are useful as benchmarks to understand the status of GWG in LMICs and to compare across countries [56]. A meta-analysis of over 1.3 million pregnancies also demonstrated that GWG outside the IOM recommendations is associated with adverse outcomes among women in Asia as well as those in the United States and Europe, lending support for applying IOM recommendations across geographic regions [57]. Nonetheless, further research is needed on context-specific GWG recommendations. Efforts are underway with a new initiative at the World Health Organization to develop global standards and recommendations for GWG based on data from diverse contexts [58].

In conclusion, prenatal BEP supplements are effective in increasing GWG and preventing inadequate weight gain during pregnancy. Continued efforts are needed to improve maternal nutrition in resource-constrained settings. Targeted BEP interventions, where BEP supplements are targeted toward pregnant women with undernutrition or inadequate weight gain, may be a promising approach to delivering BEP supplements while avoiding any potential risk of excessive weight gain. Further efforts are needed to investigate the effectiveness, cost-effectiveness, and implementation of different BEP targeting strategies.

## Supporting information

S1 Table Characteristics of the 15 studies identified but not included in the individual participant data meta-analysis of the effect of prenatal BEP supplements on GWG. (DOCX)

S2 Table PRISMA checklist for the individual participant data meta-analysis on the effects of prenatal balanced energy and protein supplements on gestational weight gain in low- and middle-income countries. (DOCX)

S3 Table Effects of prenatal balanced energy and protein supplements on gestational weight gain outcomes, after adjusting for covariates when estimating the study-specific estimates. (DOCX)

S4 Table Effects of prenatal balanced energy and protein supplements on gestational weight gain outcomes, after adjusting for covariates when estimating the study-specific estimates, using multiple imputation to impute missing covariate values. (DOCX)

S5 Table Univariate random-effects meta-regression to explore sources of heterogeneity for the effects on GWG percent adequacy at the last weight measurement. (DOCX)

S6 Table Risk of bias of the included studies. (DOCX)

S1 Fig PRISMA flow diagram for the individual participant data meta-analysis on the effects of prenatal balanced energy and protein supplements on gestational weight gain in low- and middle-income countries. IPD, individual participant data.
(DOCX)

S2 Fig Forest plot for the effect of prenatal BEP supplements on inadequate rate of GWG within the second trimester. BEP, balanced energy and protein; CI, confidence interval; GWG, gestational weight gain; T2, second trimester.
(DOCX)

S3 Fig Forest plot for the effect of prenatal BEP supplements on excessive rate of GWG within the second trimester. BEP, balanced energy and protein; CI, confidence interval; GWG, gestational weight gain; T2, second trimester.
(DOCX)

S4 Fig Forest plot for the effect of prenatal BEP supplements on inadequate rate of GWG within the third trimester. BEP, balanced energy and protein; CI, confidence interval; GWG, gestational weight gain; T3, third trimester.
(DOCX)

S5 Fig Forest plot for the effect of prenatal BEP supplements on excessive rate of GWG within the third trimester. BEP, balanced energy and protein; CI, confidence interval; GWG, gestational weight gain; T3, third trimester.
(DOCX)

## Abbreviations

BEP: balanced energy and protein
BMI: body mass index
CI: confidence interval
GWG: gestational weight gain
IOM: Institute of Medicine
LMIC: low- and middle-income country
MMS: multiple micronutrient supplement
RCT: randomized controlled trial
RR: risk ratio
SGA: small-for-gestational-age
WMD: weighted mean difference
