# Supplementary material for: The effect of prenatal balanced energy and protein supplementation on gestational weight gain: An individual participant data meta-analysis in low- and middle-income countries
Source: PLoS Med. 2025 Feb 3;22(2):e1004523. doi: 10.1371/journal.pmed.1004523 (PMC11790098; doi:10.1371/journal.pmed.1004523)
Supplement: S1 Table — (DOCX) [file pmed.1004523.s001.docx]

**S1 Table**. Characteristics of the 15 studies identified but not included in the individual participant data meta-analysis of the effect of prenatal BEP supplements on GWG^1^

| Study | Country | Intervention | Comparison | Timing of intervention initiation | Daily energy content | Forms of BEP | Delivery strategy |
| --- | --- | --- | --- | --- | --- | --- | --- |
| Lechtig 1975 [1] | Guatemala | Protein-rich gruel (*atole*) providing 163 kcal and 11.5 g protein | Nonprotein, low-energy drink (*fresco*) providing 59 kcal | During pregnancy | 163 kcal | Food rations | Untargeted |
| Mora 1979 [2] | Colombia | 60 g dried skim milk, 150 g enriched bread, and 20 g vegetable oil beginning in the third trimester. The supplement provided 856 kcal and 38.4 g protein | Normal, non-supplemented diet | From 6 months of pregnancy | 856 kcal | Food rations | Untargeted |
| Girija 1984 [3] | India | 50 g of sesame cake, 40 g of jaggery, and 10 g of oil. The supplement contributed 30 g protein and 417 kcal | No intervention | From the third trimester of pregnancy | 417 kcal | Food rations | Untargeted |
| Ross 1985 [4] | South Africa | Group 3 received a high bulk supplement, a mixture of beans and maize in a 1.2:1 ratio as mush with added vitamins. The supplement in Group 3 provided 776 kcal and 36 g vegetable protein. Group 4 received a low bulk supplementation, a porridge containing 100 g dry skimmed milk, maize flour, vitamins, and minerals. The supplement in Group 4 provided 700 kcal, 8 g vegetable protein, and 36 g animal protein. | Group 1 received placebo pills, and Group 2 received 30-90 mg of zinc gluconate daily | From 20 weeks gestation to delivery | 776 kcal (high bulk supplement); 700 kcal (low bulk supplement) | Food rations | Untargeted |
| Kardjati 1988 [5] | Indonesia | High energy beverage providing 465 kcal, 7.1 g protein, 50% fat, 10% casein, and 40% glucose, in 75 g dry mix per day | Low energy beverage providing 52 kcal, 6.2 g protein, 50% casein, 28% glucose, and 22% Textaid, in 15 g dry mix per day | From 26 to 28 weeks gestation | 465 kcal | Beverage | Untargeted |
| Ceesay 1997 [6] | The Gambia | High-energy ground biscuits provided before delivery. The biscuits contained roasted groundnuts, rice flour, sugar, and groundnut oil, and they provide a maximum possible daily intake (two biscuits) of 1016 kcal energy, 22 g protein, 56 g fat, 47 mg calcium, and 1.8 mg iron. | No intervention during pregnancy (the same high-energy ground biscuits provided after delivery) | From around 20 weeks gestation to delivery | 1016 kcal | Food rations | Untargeted |
| Potdar 2014 [7] | India | A daily snack made from green leafy vegetables, fruit, and milk provided from $\geq$90 d before pregnancy until delivery in addition to the usual diet. The snacks contained 165 kcal of energy and 10–23% of WHO Reference Nutrient Intakes of b-carotene, riboflavin, folate, vitamin B-12, calcium, and iron | A daily snack made from low-micronutrient vegetables (potato and onion) provided from $\geq$90 d before pregnancy until delivery in addition to the usual diet. The snacks contained 88 kcal and 0–7% of WHO Reference Nutrient Intakes of b-carotene, riboflavin, folate, vitamin B-12, calcium, and iron | ≥ 3 months before pregnancy | 165 kcal | Food rations | Untargeted |
| Dwarkanath 2016 [8] | India | A daily dietary supplement of 300 kcal and 15 g protein, provided as three small, round granola-type treats (*ladoos*), and made of crushed roasted peanuts, puffed rice, skimmed milk, clarified butter, and unrefined sugar. The supplement was provided from the first trimester of pregnancy to delivery | Habitual diet with no supplement | From 12 $\pm$ 1 weeks gestation to delivery | 300 kcal | Food rations | Untargeted |
| Devi 2017 [9] | India | 1. 500 mL of milk/d plus a 10-mg vitamin B-12 tablet/d  2. Milk of 500 mL/d plus a placebo tablet.  The 500 mL milk supplement in both arms provided 320 kcal energy and 16.5 g protein, equivalent to a 21% protein-energy ratio | A placebo tablet only | From 11 $\pm$ 2 weeks gestation to delivery | 320 kcal | Beverage | Untargeted |
| Mantaring 2018 [10] | Philippines | A beverage supplement providing 140 kcal and 7.9 g protein per serving, multivitamin/ minerals, enriched or not with the probiotics *Lactobacillus rhamnosus* and *Bifidobacterium lactis*, from the third trimester of pregnancy until at least two months post-delivery | No intervention | From 24-28 weeks of gestation to 2 months after delivery | 140 kcal | Beverage | Untargeted |
| Olney 2018 [11] | Guatemala | Permutation of varying family ration sizes and individual ration types:  Full family ration (rice, beans, oil) + CSB  Reduced family ration + CSB  No family ration + CSB  Full family ration + LNS  Full family ration + MNP | No intervention | From pregnancy to up to 6 months postpartum | 270 kcal/d per capita from family ration; 118 kcal from LNS | Food rations | Untargeted |
| Stevens 2018 [12] | Bangladesh | The supplement consisted of 27% pigeon pea, 35% banana, 16% sugar, 9% peanuts, 6% whole milk powder, 6% sesame seeds and 1% iodized salt. The serving size was 173 g, providing 522 kcal, 19.5 g protein, 15.8 g fat, and various vitamins and minerals | No supplement | During pregnancy | 522 kcal | Food rations | Targeted |
| Tabrizi 2019 [13] | Iran | The food supplement was given every 2 months and included 10 kg of rice, 1400 g of pasta, 5 kg of frozen chicken, 6 cans of tuna, 4 kg of lentils, 2 kg of soybeans, 2 kg packaged palm, 2 kg packaged cheese, 2.5 kg of oil, 1 kg of honey, and 2 kg of sugar | No supplement | From 10 weeks gestation to delivery | 1500 kcal | Food rations | Targeted |
| Lee, 2022 [14] | Ethiopia | 1. Enhanced nutrition package with placebo infection control tablet. The enhanced nutrition package included daily MMS; participants with MUAC <23 cm received daily BEP in the form of CSB (SuperCereal) providing 760 kcal and 28 g protein daily  2. Enhanced nutrition package with azithromycin  3. Enhanced nutrition package with enhanced infection management package | 1. Routine care with placebo infection control tablet  2. Routine care with azithromycin  3. Routine care with enhanced infection management package | From < 24 weeks gestation to delivery | 760 kcal | Food rations | Targeted |
| NCT03558464 | Kenya | LNS (1 sachet/day), anthelminthic treatment (mebendazole once during the second trimester), soap and chlorine solution for the woman and the household, and agricultural training | Agricultural training | From ≤ 20 weeks gestation to up to 6 months postpartum | Unclear | Lipid-based supplement | Untargeted |

^1^ BMI, body mass index; BEP, balanced energy and protein; CSB, corn-soy blend; GA, gestational age; GWG, gestational weight gain; IFA, iron and folic acid; IOM, Institute of Medicine; LMP, last menstrual period; LNS, lipid-based nutrient supplements; MMN, multiple micronutrients; MMS, multiple micronutrient supplements; MNP, micronutrient powder; MUAC, mid-upper arm circumference; PLA, participatory learning and action; RDA, recommended daily allowance; RUSF, ready-to-use supplemental food; SP, sulfadoxine-pyrimethamine; UNIMMAP, UNICEF/WHO/United Nations multiple micronutrient supplements for pregnant and lactating women; WaSH, water, sanitation and hygiene; WHO, World Health Organization.

**REFERENCES**

1. Lechtig A, Habicht JP, Delgado H, Klein RE, Yarbrough C, Martorell R. Effect of food supplementation during pregnancy on birthweight. Pediatrics. 1975;56(4):508-20. PubMed PMID: 1165955.

2. Mora JO, de Paredes B, Wagner M, de Navarro L, Suescun J, Christiansen N, et al. Nutritional supplementation and the outcome of pregnancy. I. Birth weight. Am J Clin Nutr. 1979;32(2):455-62. doi: 10.1093/ajcn/32.2.455. PubMed PMID: 420135.

3. Girija A, Geervani P, Rao GN. Influence of dietary supplementation during pregnancy on lactation performance. J Trop Pediatr. 1984;30(2):79-83. doi: 10.1093/tropej/30.2.79. PubMed PMID: 6726837.

4. Ross SM, Nel E, Naeye RL. Differing effects of low and high bulk maternal dietary supplements during pregnancy. Early Hum Dev. 1985;10(3-4):295-302. doi: 10.1016/0378-3782(85)90061-1. PubMed PMID: 2985353.

5. Kardjati S, Kusin JA, De With C. Energy supplementation in the last trimester of pregnancy in East Java: I. Effect on birthweight. Br J Obstet Gynaecol. 1988;95(8):783-94. doi: 10.1111/j.1471-0528.1988.tb06553.x. PubMed PMID: 3048373.

6. Ceesay SM, Prentice AM, Cole TJ, Foord F, Weaver LT, Poskitt EM, et al. Effects on birth weight and perinatal mortality of maternal dietary supplements in rural Gambia: 5 year randomised controlled trial. Bmj. 1997;315(7111):786-90. doi: 10.1136/bmj.315.7111.786. PubMed PMID: 9345173; PubMed Central PMCID: PMCPMC2127544.

7. Potdar RD, Sahariah SA, Gandhi M, Kehoe SH, Brown N, Sane H, et al. Improving women's diet quality preconceptionally and during gestation: effects on birth weight and prevalence of low birth weight--a randomized controlled efficacy trial in India (Mumbai Maternal Nutrition Project). Am J Clin Nutr. 2014;100(5):1257-68. Epub 20140917. doi: 10.3945/ajcn.114.084921. PubMed PMID: 25332324; PubMed Central PMCID: PMCPMC4196482.

8. Dwarkanath P, Hsu JW, Tang GJ, Anand P, Thomas T, Thomas A, et al. Energy and Protein Supplementation Does Not Affect Protein and Amino Acid Kinetics or Pregnancy Outcomes in Underweight Indian Women. J Nutr. 2016;146(2):218-26. Epub 20160113. doi: 10.3945/jn.115.218776. PubMed PMID: 26764317.

9. Devi S, Mukhopadhyay A, Dwarkanath P, Thomas T, Crasta J, Thomas A, et al. Combined Vitamin B-12 and Balanced Protein-Energy Supplementation Affect Homocysteine Remethylation in the Methionine Cycle in Pregnant South Indian Women of Low Vitamin B-12 Status. J Nutr. 2017;147(6):1094-103. Epub 20170426. doi: 10.3945/jn.116.241042. PubMed PMID: 28446631.

10. Mantaring J, Benyacoub J, Destura R, Pecquet S, Vidal K, Volger S, et al. Effect of maternal supplement beverage with and without probiotics during pregnancy and lactation on maternal and infant health: a randomized controlled trial in the Philippines. BMC Pregnancy Childbirth. 2018;18(1):193. Epub 20180531. doi: 10.1186/s12884-018-1828-8. PubMed PMID: 29855271; PubMed Central PMCID: PMCPMC5984298.

11. Olney DK, Leroy J, Bliznashka L, Ruel MT. PROCOMIDA, a Food-Assisted Maternal and Child Health and Nutrition Program, Reduces Child Stunting in Guatemala: A Cluster-Randomized Controlled Intervention Trial. J Nutr. 2018;148(9):1493-505. doi: 10.1093/jn/nxy138. PubMed PMID: 30184223; PubMed Central PMCID: PMCPMC6118165.

12. Stevens B, Watt K, Brimbecombe J, Clough A, Judd JA, Lindsay D. A village-matched evaluation of providing a local supplemental food during pregnancy in rural Bangladesh: a preliminary study. BMC Pregnancy Childbirth. 2018;18(1):286. Epub 20180704. doi: 10.1186/s12884-018-1915-x. PubMed PMID: 29973170; PubMed Central PMCID: PMCPMC6030796.

13. Tabrizi JS, Asghari A, Pourali F, Kousha H, Nikniaz L. Effects of Food Supplementation During Pregnancy on Maternal Weight Gain, Hemoglobin Levels and Pregnancy Outcomes in Iran. Matern Child Health J. 2019;23(2):258-64. doi: 10.1007/s10995-018-2648-1. PubMed PMID: 30569304.

14. Lee AC, Abate FW, Mullany LC, Baye E, Berhane YY, Derebe MM, et al. Enhancing Nutrition and Antenatal Infection Treatment (ENAT) study: protocol of a pragmatic clinical effectiveness study to improve birth outcomes in Ethiopia. BMJ Paediatr Open. 2022;6(1). doi: 10.1136/bmjpo-2021-001327. PubMed PMID: 36053580; PubMed Central PMCID: PMCPMC8762145.
