# Supplementary material for: The effect of prenatal balanced energy and protein supplementation on gestational weight gain: An individual participant data meta-analysis in low- and middle-income countries
Source: PLoS Med. 2025 Feb 3;22(2):e1004523. doi: 10.1371/journal.pmed.1004523 (PMC11790098; doi:10.1371/journal.pmed.1004523)
Supplement: S3 Table — (DOCX) [file pmed.1004523.s003.docx]

**S3 Table.** Effects of prenatal balanced energy and protein supplements on gestational weight gain outcomes, after adjusting for covariates when estimating the study-specific estimates^1^

|  | GWG percent adequacy at the last gestational weight measurement | Estimated total GWG at delivery | Severely inadequate GWG | Inadequate GWG | Excessive GWG |
| --- | --- | --- | --- | --- | --- |
|  | Mean difference (95% CI) | Mean difference (95% CI) | RR (95% CI) | RR (95% CI) | RR (95% CI) |
| Kaseb, 2002 | 21.14 (-5.66, 47.95) | NA^2^ | 0.71 (0.20, 2.47) | 0.77 (0.35, 1.70) | 1.05 (0.52, 2.09) |
| Huybregts, 2009 | 6.64 (2.46, 10.81) | 0.74 (0.23, 1.25) | 0.92 (0.85, 1.00) | 0.97 (0.93, 1.02) | 1.00 (0.50, 1.97) |
| Moore, 2012 | 4.33 (-2.71, 11.36) | 0.29 (-0.43, 1.02) | 0.99 (0.88, 1.11) | 1.00 (0.94, 1.06) | 1.21 (0.76, 1.94) |
| Saville, 2018 | 1.19 (-1.05, 3.43) | 0.09 (-0.18, 0.37) | 1.01 (0.89, 1.15) | 0.97 (0.93, 1.02) | 1.55 (0.89, 2.71) |
| Hambidge, 2019 | 4.73 (-4.20, 13.66) | 0.49 (-0.60, 1.58) | 0.91 (0.81, 1.01) | 0.97 (0.89, 1.05) | 0.85 (0.63, 1.14) |
| Neufeld, 2019 | -1.77 (-10.04, 6.51) | -0.02 (-0.86, 0.83) | 0.84 (0.36, 1.94) | 0.94 (0.61, 1.44) | 1.01 (0.77, 1.32) |
| Khan, 2021 | 2.49 (-2.00, 6.97) | 0.35 (-0.16, 0.86) | 0.93 (0.81, 1.07) | 0.94 (0.89, 0.99) | 1.17 (0.92, 1.49) |
| Taneja, 2022 | 18.44 (14.06, 22.83) | 2.03 (1.58, 2.47) | 0.68 (0.61, 0.76) | 0.80 (0.74, 0.86) | 1.72 (1.41, 2.10) |
| de Kok, 2022 | 4.46 (0.55, 8.36) | 0.61 (0.21, 1.02) | 0.96 (0.87, 1.05) | 0.97 (0.91, 1.03) | 1.21 (0.89, 1.64) |
| Muhammad, 2022 | 7.64 (-1.72, 16.99) | 0.87 (-0.18, 1.92) | 0.93 (0.80, 1.08) | 0.99 (0.93, 1.06) | 0.93 (0.60, 1.43) |
| Erchick, 2023 | 3.98 (-1.98, 9.94) | 0.48 (-0.26, 1.23) | 0.92 (0.79, 1.08) | 0.93 (0.84, 1.03) | 0.99 (0.65, 1.50) |

^1^ Value are mean differences for continuous outcomes and risk ratios for binary outcomes with 95% confidence intervals comparing prenatal balanced energy and protein supplements to control. The covariates included maternal age, maternal years of education, parity, gestational age at enrollment, maternal height, pre-pregnancy or early-pregnancy BMI, and hemoglobin concentration at enrollment, all as continuous variables. Availability of these covariates varied across studies, and the covariates available in the study were adjusted. CI, confidence interval; GWG, gestational weight gain; RR, risk ratio.

^2^ Could not be estimated due to missing information on gestational age at delivery.
