# Supplementary material for: The effect of prenatal balanced energy and protein supplementation on gestational weight gain: An individual participant data meta-analysis in low- and middle-income countries
Source: PLoS Med. 2025 Feb 3;22(2):e1004523. doi: 10.1371/journal.pmed.1004523 (PMC11790098; doi:10.1371/journal.pmed.1004523)
Supplement: S4 Table — (DOCX) [file pmed.1004523.s004.docx]

**S4 Table.** Effects of prenatal balanced energy and protein supplements on gestational weight gain outcomes, after adjusting for covariates when estimating the study-specific estimates, using multiple imputation to impute missing covariate values^1^

|  | GWG percent adequacy at the last gestational weight measurement | Estimated total GWG at delivery | Severely inadequate GWG | Inadequate GWG | Excessive GWG |
| --- | --- | --- | --- | --- | --- |
|  | Mean difference (95% CI) | Mean difference (95% CI) | RR (95% CI) | RR (95% CI) | RR (95% CI) |
| Kaseb, 2002 | 20.35 (-7.61, 48.30) | NA^2^ | 0.69 (0.19, 2.57) | 0.72 (0.31, 1.67) | 1.60 (0.73, 3.48) |
| Huybregts, 2009 | 6.30 (2.20, 10.39) | 0.68 (0.18, 1.18) | 0.92 (0.85, 1.00) | 0.94 (0.90, 0.99) | 1.04 (0.54, 2.02) |
| Moore, 2012 | 4.01 (-2.93, 10.94) | 0.30 (-0.42, 1.01) | 0.99 (0.88, 1.11) | 0.99 (0.92, 1.07) | 1.28 (0.81, 2.04) |
| Saville, 2018 | 1.48 (-0.70, 3.66) | 0.13 (-0.13, 0.40) | 1.00 (0.90, 1.11) | 0.98 (0.94, 1.02) | 1.63 (1.12, 2.36) |
| Hambidge, 2019 | 4.37 (-3.90, 12.64) | 0.43 (-0.66, 1.51) | 0.91 (0.81, 1.02) | 0.96 (0.87, 1.05) | 0.85 (0.65, 1.09) |
| Neufeld, 2019 | -1.63 (-10.49, 7.22) | -0.05 (-0.93, 0.83) | 0.91 (0.40, 2.06) | 0.95 (0.68, 1.33) | 0.92 (0.71, 1.18) |
| Khan, 2021 | 2.48 (-1.94, 6.89) | 0.33 (-0.17, 0.83) | 0.96 (0.85, 1.07) | 0.95 (0.93, 0.98) | 1.13 (0.85, 1.51) |
| Taneja, 2022 | 17.78 (13.52, 22.04) | 1.99 (1.56, 2.42) | 0.69 (0.62, 0.77) | 0.79 (0.73, 0.85) | 1.72 (1.42, 2.09) |
| de Kok, 2022 | 4.49 (0.58, 8.39) | 0.62 (0.21, 1.03) | 0.96 (0.87, 1.05) | 0.94 (0.88, 1.00) | 1.18 (0.87, 1.60) |
| Muhammad, 2022 | 6.54 (-2.60, 15.67) | 0.78 (-0.33, 1.90) | 0.93 (0.79, 1.09) | 0.95 (0.86, 1.06) | 1.00 (0.66, 1.49) |
| Erchick, 2023 | 3.88 (-1.99, 9.75) | 0.48 (-0.25, 1.22) | 0.94 (0.80, 1.09) | 0.93 (0.84, 1.03) | 0.94 (0.61, 1.43) |

^1^ Value are mean differences for continuous outcomes and risk ratios for binary outcomes with 95% confidence intervals comparing prenatal balanced energy and protein supplements to control. The covariates included maternal age, maternal years of education, parity, gestational age at enrollment, maternal height, pre-pregnancy or early-pregnancy BMI, and hemoglobin concentration at enrollment, all as continuous variables. Missing data on covariates were imputed using multiple imputation. CI, confidence interval; GWG, gestational weight gain; RR, risk ratio.

^2^ Could not be estimated due to missing information on gestational age at delivery.
