# Supplementary material for: The effect of prenatal balanced energy and protein supplementation on gestational weight gain: An individual participant data meta-analysis in low- and middle-income countries
Source: PLoS Med. 2025 Feb 3;22(2):e1004523. doi: 10.1371/journal.pmed.1004523 (PMC11790098; doi:10.1371/journal.pmed.1004523)
Supplement: S5 Table — (DOCX) [file pmed.1004523.s005.docx]

**S5 Table.** Univariate random-effects meta-regression to explore sources of heterogeneity for the effects on GWG percent adequacy at the last weight measurement^1^

|  | GWG percent adequacy at the last weight measurement | | | Estimated total GWG at delivery | | | Severely inadequate GWG | | | Inadequate GWG | | | Excessive GWG | | |
| --- | --- | --- | --- | --- | --- | --- | --- | --- | --- | --- | --- | --- | --- | --- | --- |
| Study-level characteristic | Mean difference (95% CI) | *P* | % total between-study variance explained by variable | Mean difference (95% CI) | *P* | % total between-study variance explained by variable | RR (95% CI) | *P* | % total between-study variance explained by variable | RR (95% CI) | *P* | % total between-study variance explained by variable | RR (95% CI) | *P* | % total between-study variance explained by variable |
| Geographic area^2^ |  | | 0% |  | | 0% |  | | 0% |  | | 0% |  | | 0% |
| Latin America and the Caribbean | *Ref* | *Ref* |  | *Ref* | *Ref* |  | *Ref* | *Ref* |  | *Ref* | *Ref* |  | *Ref* | *Ref* |  |
| Middle East and North Africa | -2.21 (-43.29, 38.87) | 0.91 |  | NA | NA |  | 0.90 (0.21, 3.79) | 0.87 |  | 1.03 (0.39, 2.72) | 0.94 |  | 0.98 (0.33, 2.94) | 0.97 |  |
| South Asia | -3.85 (-17.82, 10.13) | 0.55 |  | -0.04 (-1.67, 1.59) | 0.96 |  | 1.17 (0.84, 1.64) | 0.32 |  | 1.12 (0.92, 1.36) | 0.21 |  | 1.06 (0.60, 1.88) | 0.83 |  |
| Sub-Saharan Africa | -5.16 (-20.41, 10.08) | 0.46 |  | -0.21 (-2.02, 1.61) | 0.80 |  | 1.25 (0.88, 1.78) | 0.19 |  | 1.15 (0.94, 1.41) | 0.15 |  | 1.11 (0.57, 2.17) | 0.74 |  |
| Energy content of BEP supplements, 100 kcal/d | 1.51 (-0.10, 3.11) | 0.06 | 49% | 0.18 (-0.02, 0.39) | 0.08 | 42% | 0.97 (0.93, 1.01) | 0.16 | 31% | 0.98 (0.96, 1.01) | 0.13 | 26% | 1.08 (1.03, 1.13) | 0.008 | 96% |
| Forms of BEP |  | | 0% |  | | 0% |  | | 24% |  | | 0% |  | | 21% |
| Food ration | *Ref* | *Ref* |  | *Ref* | *Ref* |  | *Ref* | *Ref* |  | *Ref* | *Ref* |  | *Ref* | *Ref* |  |
| Lipid-based supplement | -2.00 (-11.37, 7.38) | 0.64 |  | -0.10 (-1.31, 1.12) | 0.86 |  | 1.11 (0.92, 1.35) | 0.25 |  | 1.06 (0.95, 1.17) | 0.26 |  | 0.81 (0.56, 1.15) | 0.20 |  |
| Delivery strategies of BEP |  | | 99% |  | | 86% |  | | 90% |  | | 88% |  | | 32% |
| Untargeted | *Ref* | *Ref* |  | *Ref* | *Ref* |  | *Ref* | *Ref* |  | *Ref* | *Ref* |  | *Ref* | *Ref* |  |
| Targeted | 12.40 (7.32, 17.48) | 0.0004 |  | 1.36 (0.57, 2.15) | 0.004 |  | 0.78 (0.68, 0.89) | 0.002 |  | 0.87 (0.80, 0.95) | 0.005 |  | 1.20 (0.81, 1.77) | 0.32 |  |
| Mean pre-pregnancy or early-pregnancy BMI, kg/m^2^ | 1.19 (-1.40, 3.79) | 0.33 | 30% | 0.03 (-0.31, 0.37) | 0.84 | 7% | 0.93 (0.86, 1.01) | 0.08 | 36% | 0.97 (0.93, 1.01) | 0.15 | 23% | 0.99 (0.88, 1.11) | 0.84 | 0% |

^1^ BEP, balanced energy and protein; BMI, body mass index; CI, confidence interval; GWG, gestational weight gain; RR, risk ratio.

^2^ For the Women First Study, a multi-country study conducted in Guatemala, India, and Pakistan, the country-specific estimates were calculated and used in the corresponding category of geographic area.
