# Supplementary material for: The effect of prenatal balanced energy and protein supplementation on gestational weight gain: An individual participant data meta-analysis in low- and middle-income countries
Source: PLoS Med. 2025 Feb 3;22(2):e1004523. doi: 10.1371/journal.pmed.1004523 (PMC11790098; doi:10.1371/journal.pmed.1004523)
Supplement: S1 Fig — IPD, individual participant data. (DOCX) [file pmed.1004523.s007.docx]

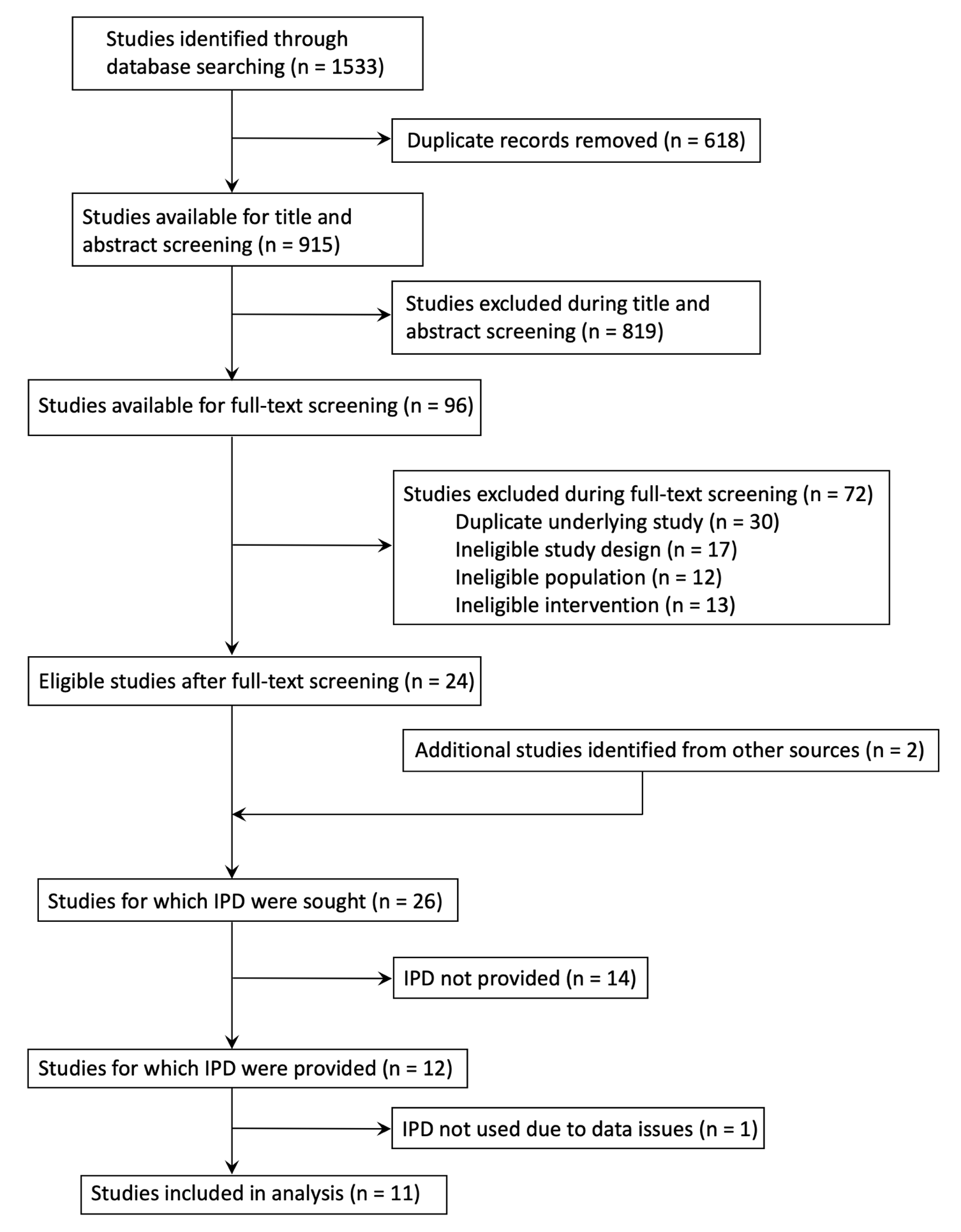


**S1 Fig.** PRISMA flow diagram for the individual participant data meta-analysis on the effects of prenatal balanced energy and protein supplements on gestational weight gain in low- and middle-income countries. IPD, individual participant data.
