# Supplementary material for: The effect of prenatal balanced energy and protein supplementation on gestational weight gain: An individual participant data meta-analysis in low- and middle-income countries
Source: PLoS Med. 2025 Feb 3;22(2):e1004523. doi: 10.1371/journal.pmed.1004523 (PMC11790098; doi:10.1371/journal.pmed.1004523)
Supplement: S5 Fig — BEP, balanced energy and protein; CI, confidence interval; GWG, gestational weight gain; T3, third trimester. (DOCX) [file pmed.1004523.s011.docx]

**
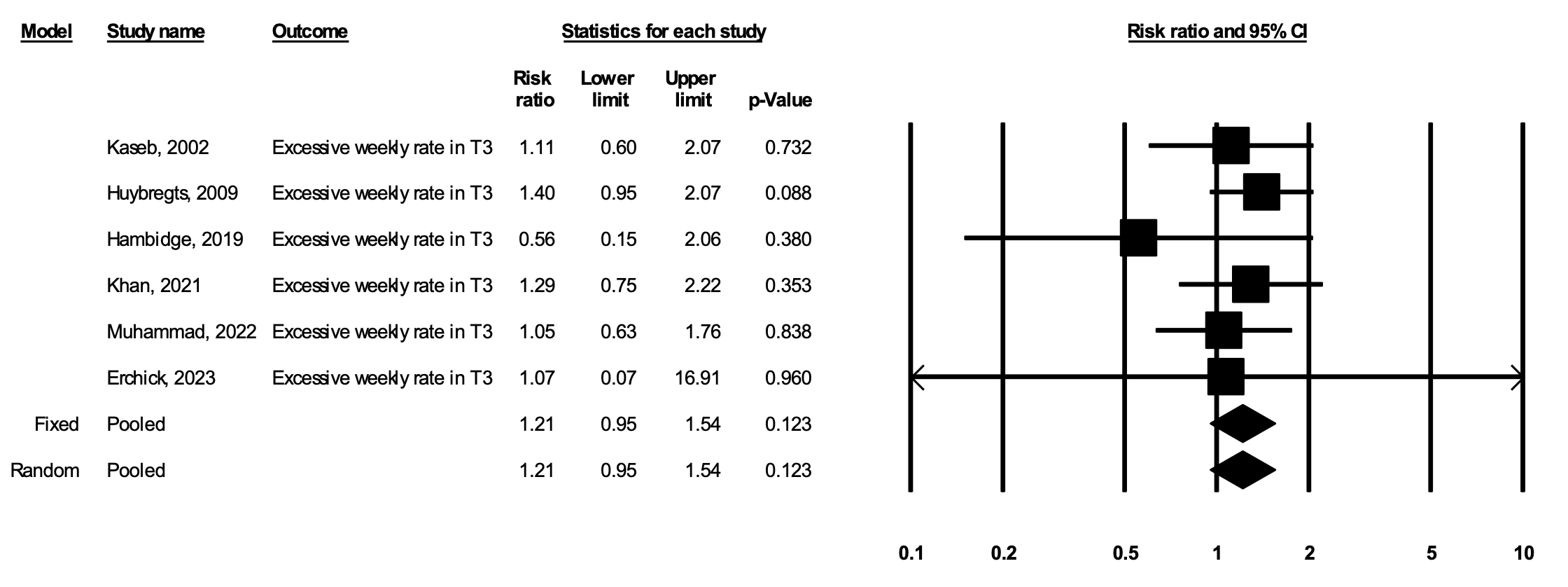
**

**S5 Fig.** Forest plot for the effect of prenatal BEP supplements on excessive rate of GWG within the third trimester. BEP, balanced energy and protein; CI, confidence interval; GWG, gestational weight gain; T3, third trimester.
